# Functional variation among LPMOs revealed by the inhibitory effects of cyanide and buffer ions

**DOI:** 10.1002/1873-3468.15105

**Published:** 2025-02-06

**Authors:** Ole Golten, Lorenz Schwaiger, Zarah Forsberg, Kelsi R. Hall, Anton A. Stepnov, Tom Z. Emrich‐Mills, Iván Ayuso‐Fernández, Morten Sørlie, Roland Ludwig, Åsmund Kjendseth Røhr, Vincent G. H. Eijsink

**Affiliations:** ^1^ Faculty of Chemistry, Biotechnology and Food Science Norwegian University of Life Sciences (NMBU) Ås Norway; ^2^ Department of Food Science and Technology, Institute of Food Technology University of Natural Resources and Life Sciences Vienna Austria; ^3^ School of Biological Sciences University of Canterbury Christchurch New Zealand; ^4^ Biotechnology Department Margarita Salas Center for Biological Research (CIB‐CSIC) Madrid Spain

**Keywords:** cellulose, chitin, copper, cyanide, LPMO, lytic polysaccharide monooxygenase

## Abstract

Enzymes known as lytic polysaccharide monooxygenases (LPMOs) are mono‐copper polysaccharide‐degrading peroxygenases that engage in several on‐ and off‐pathway redox reactions involving O_2_ and H_2_O_2_. Herein, we show that the known metalloenzyme inhibitor cyanide inhibits reductive activation of LPMOs by binding to the LPMO‐Cu(II) state and that the degree of inhibition depends on the concentrations of the polysaccharide substrate, the reductant and H_2_O_2_. Importantly, this analysis revealed differences between fungal *Nc*AA9C and bacterial *Sm*AA10A, which have different secondary copper coordination spheres. These differences were also highlighted by the observation that phosphate, a commonly used buffer ion, strongly inhibits *Nc*AA9C while not affecting reactions with *Sm*AA10A. The results provide insight into LPMO inhibition and catalysis and highlight pitfalls in the analysis thereof.

## Graphical abstract

This study addresses the inhibition of lytic polysaccharide monooxygenases (LPMOs) by cyanide and explains how and why the magnitude of observed inhibitory effects depends on the way LPMO reactions are setup and on the type of LPMO.

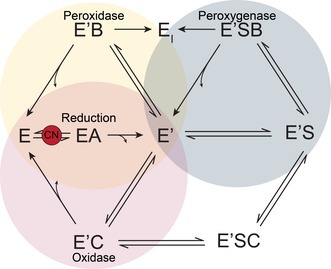

## Abbreviations

**AA**, auxiliary activity

**Bis‐Tris**, 2‐[Bis‐(2‐hydroxyethyl)‐amino]‐2‐hydroxymethyl‐propane‐1,3‐diol

**CAZy**, Carbohydrate‐active enzyme database

**EPR**, Electron paramagnetic resonance

**KMSA**, potassium methanesulfonate

**LPMO**, lytic polysaccharide monooxygenase

**MOPS**, 3‐N(morpholino)propanesulfonic acid

**SHE**, standard hydrogen electrode

**Tris/HCl**, Tris(hydroxylmethyl)aminomethane hydrochloride

Lytic polysaccharide monooxygenases (LPMOs) are mono‐nuclear copper enzymes capable of activating C‐H bonds with bond dissociation energies close to 100 kcal·mol^−1^ [1] and have received substantial interest due to their fascinating chemistry [2, 3] and relevance in industry [4, 5, 6]. Since their discovery [7, 8], LPMOs have been identified in several phyla of life and their (putative) roles today include roles in biomass degradation [9, 10], bacterial, fungal, and oomycete pathogenicity [11, 12, 13, 14], insect molting [15] and bacterial cell wall remodeling [16]. The copper ion is coordinated by a highly conserved histidine brace [2, 17], while amino acids in the immediate vicinity of this brace, i.e., the second coordination sphere, tune copper reactivity [18, 19, 20, 21]. Currently, based on their sequences, LPMOs are classified into eight families of Auxiliary Activities (AA) in the carbohydrate‐active enzyme database (CAZy) [22].

**Fig. 1:**
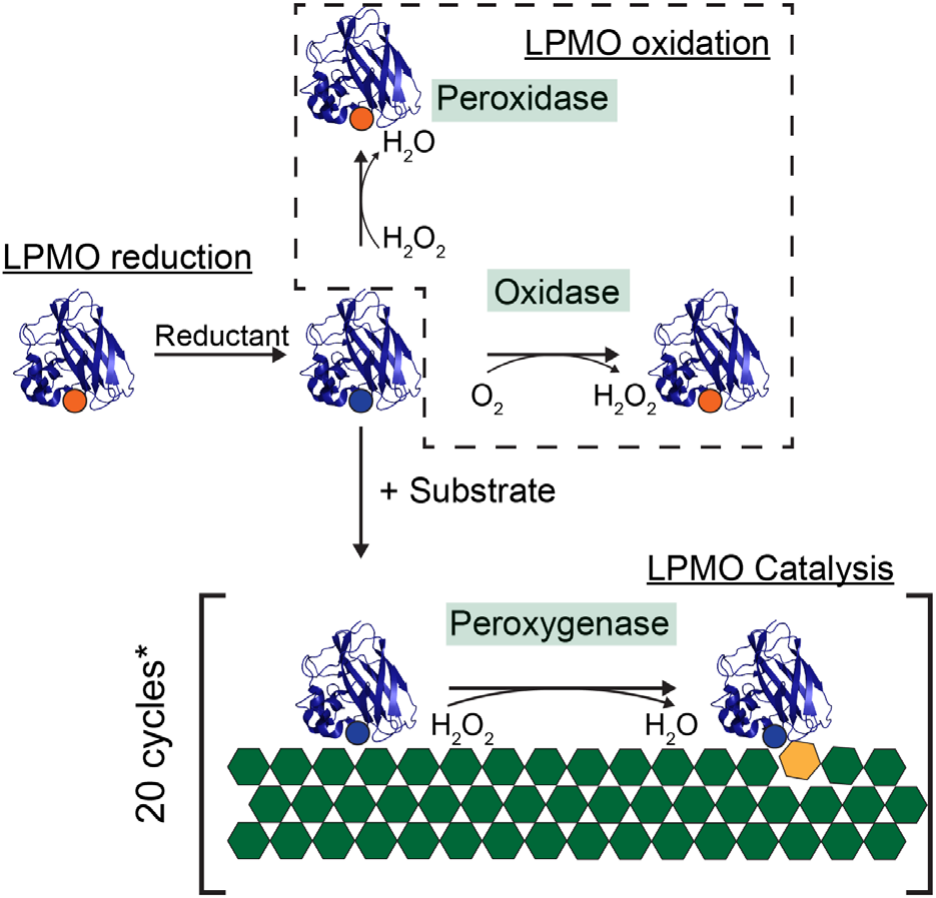
LPMO reactions. First, reduction from Cu(II) (orange) to Cu(I) (blue) is required for all subsequent reactions. Without a polymeric substrate, the LPMO can react with H_2_O_2_ in the peroxidase reaction or with O_2_ in the oxidase reaction; in both cases, the LPMO becomes oxidized and requires a new reduction event. In the presence of a polymeric substrate and H_2_O_2_, LPMO catalysis occurs, yielding oxidative cleavage of the substrate. Figure was adapted from [31]. *Currently available data typically show that, with suitable substrates and controlled reaction conditions, LPMOs on average catalyze ~ 20 cleavages before requiring a new reduction event [27, 32].

While LPMOs show functional variation, for example, in terms of substrate specificity and oxidative regioselectivity [7, 23, 24, 25, 26, 27, 28, 29, 30], they all appear to engage in a similar set of interrelated on‐ and off‐pathway reactions (Fig. 1). It has been challenging to unravel these reactions, partly due to initial confusion surrounding the co‐substrate. Initially, LPMOs were thought to be monooxygenases ($R-H+O_{2}+2e^{-}+2H^{+}\to R-\mathrm{OH}+2H_{2}O$), using O_2_ as a co‐substrate [7, 17, 33]. However, recent work has shown that LPMOs are peroxygenases ($R-H+H_{2}O_{2}\to R-\mathrm{OH}+H_{2}O$) using H_2_O_2_ as the co‐substrate [27, 34, 35, 36, 37, 38]. Confusingly, in reactions run under “monooxygenase conditions” (i.e., LPMO + substrate + reductant, aerobic conditions), hereafter referred to as *in situ* H_2_O_2_‐limiting conditions, H_2_O_2_ is generated *in situ* through the LPMO oxidase activity [39] and abiotic oxidation of the reductant [40, 41]. Thus, it has been claimed that most, if not all, observed apparent monooxygenase reactions, are peroxygenase reactions limited by the rate of *in situ* generation of H_2_O_2_.

Analysis of LPMO catalysis is further complicated by the insoluble nature of the substrate, autocatalytic oxidative enzyme inactivation [34], and the effects of free transition metals. Free copper is particularly problematic because its presence will strongly affect the abiotic oxidation of commonly used reductants such as ascorbate, thus affecting the *in situ* generation of H_2_O_2_ [41]. In addition, oxidative damage to LPMOs will lead to the release of copper from the active site, meaning that free copper levels, and thus the levels of *in situ* generated H_2_O_2_, vary as the reaction proceeds [41, 42]. The combination of the latter two effects can make LPMO inactivation a self‐reinforcing process [43]. Finally, the contribution of the oxidase reaction to the *in situ* generation of H_2_O_2_ varies between LPMOs [44]. Running reactions with externally added H_2_O_2_ or controlled *in situ* generation of H_2_O_2_ with enzymes like glucose oxidase can overcome these complications to some extent [6, 27, 34].

The issues discussed above are also relevant when assessing LPMO inhibitors, while, at the same time, the use of inhibitors can shed light on LPMO catalysis. So far, little is known about LPMO inhibition, and available studies have focused on natural organic inhibitors [45, 46], organic acids, and amino acids [47]. The solvent‐exposed copper can be affected by known inhibitors of copper enzymes, such as cyanide [48, 49], and, indeed, early studies done before the discovery of the peroxygenase activity of LPMOs showed that cyanide inhibits the bacterial chitin‐active LPMO *Sm*AA10A [7]. Cyanide is an interesting LPMO inhibitor since Cu‐CN complexes may mimic some of the possible copper‐oxo species generated during on‐ or off‐pathway catalysis, in particular, the Cu‐superoxide complex that likely emerges when a reduced LPMO reacts with molecular oxygen [50]. Such mimicking is less likely for the peroxygenase reaction, which depends on the homolytic cleavage of H_2_O_2_ at the Cu(I) active site, yielding a copper‐hydroxide (Cu(II)‐OH) and a hydroxyl radical, OH^•^. While the next reaction step remains enigmatic, it has been repeatedly proposed that a copper‐oxyl (Cu(II)‐O^•^) is formed, which performs the hydrogen atom abstraction from the substrate [18, 35, 51, 52].

**Fig. 2:**
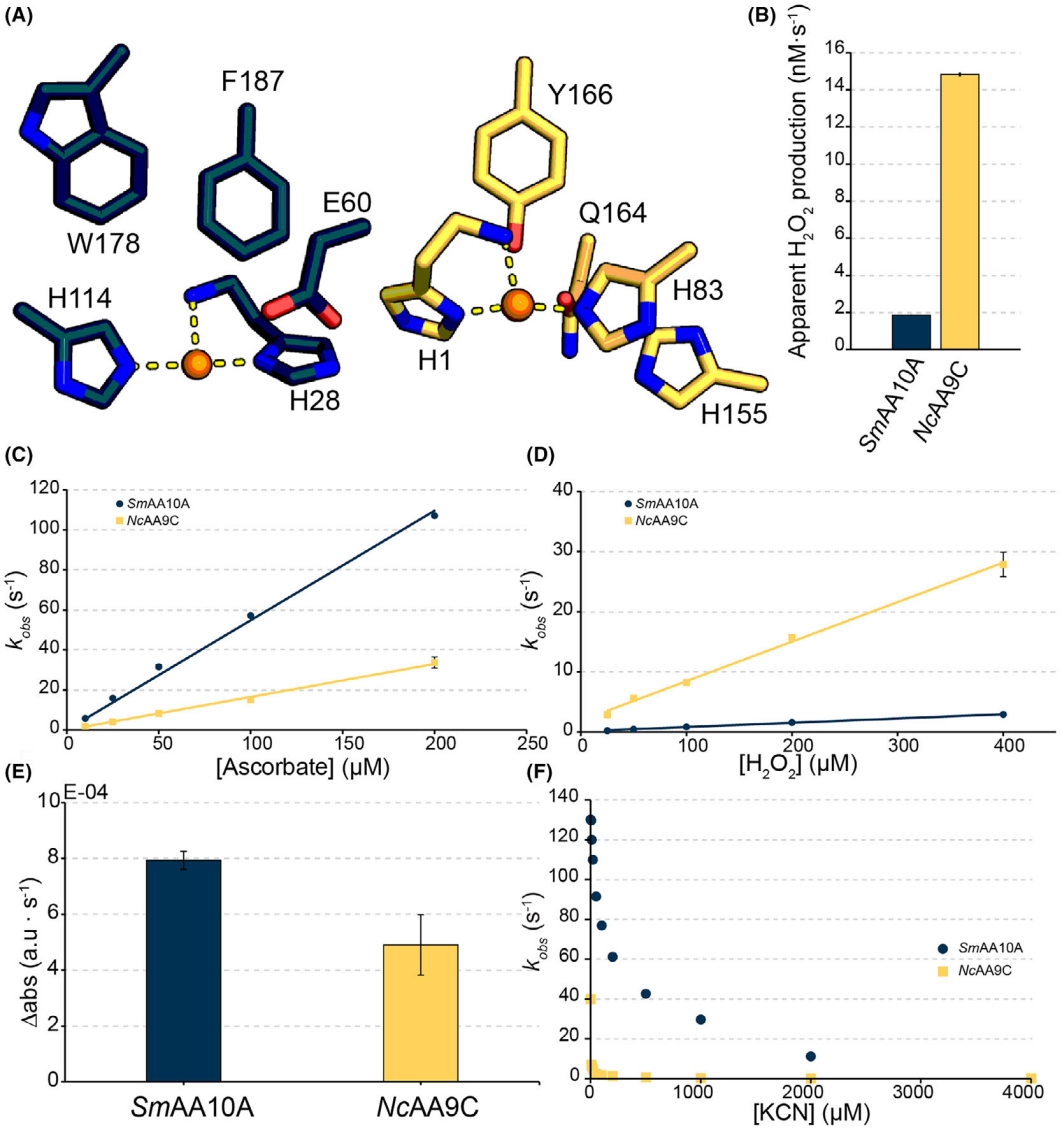
Comparison of copper reactivity in *Sm*AA10A and *Nc*AA9C. (A) Structural comparison of the active sites of *Sm*AA10A and *Nc*AA9C showing the conserved histidine brace and selected second sphere residues. (B) Oxidase activity of 1 μm *Sm*AA10A and *Nc*AA9C reacting with 1 mm ascorbate at 30 °C. (C) Pseudo‐first‐order reduction rates (*k*_obs_) with increasing concentrations of ascorbate (10–200 μm). (D) Pseudo‐first‐order LPMO oxidation rates (*k*_obs_) with increasing concentrations of H_2_O_2_ (25–400 μm). (E) Initial rates of 2,6‐DMP oxidation in reactions with 2 μm enzyme, 1 mm 2,6‐DMP and 50 μm H_2_O_2_. All reactions were done in 50 mm MOPS, pH 7.0, in triplicates. Standard deviations are shown as error bars. (F) Pseudo‐first‐order reduction rates (*k*_obs_) in reactions with 200 μm ascorbate and varying amounts of cyanide (0–4000 μm). The increased apparent reduction rate observed in the reaction with 200 μm ascorbate and no cyanide (compared to panel C) results from the increased instrument dead time in the required double mixing setup, meaning that the entire single exponential cannot be measured.

In this work, we have probed the effect of cyanide on LPMOs during on‐ and off‐pathway turnover. By gauging the effect of cyanide on both the productive reaction and off‐pathway reactions, we provide insight into the impact of substrate and reductant concentrations, and we unveil principles and pitfalls of interpreting LPMO reaction kinetics. Importantly, our studies, which include an assessment of the impact of buffer ions, reveal important differences between the two studied LPMOs, fungal *Nc*AA9C and bacterial *Sm*AA10A (Fig. 2A). To the best of our knowledge, this work is the first LPMO inhibition study that assesses inhibitory effects on product formation in reactions with natural LPMO substrates over time.

## Materials and methods

### Materials

All chemicals were sourced from Sigma‐Aldrich if not stated otherwise. β‐chitin was sourced from France Chitin, Orange, France, batch number 20140101 and milled to a particle size of 75–200 μm using a PM 200 planetary ball mill (Retsch, Haan, Germany) equipped with zirconium oxide milling tools. Aliquots of ascorbate were prepared at a 100 mm concentration in TraceSELECT™ water (Honeywell, Charlotte, NC, USA) before storing at −20 °C until use. All reactions containing cyanide were prepared using potassium cyanide. Cyanide stocks were prepared fresh by dissolving potassium cyanide in 0.5 m 3‐N(morpholino)propanesulfonic acid (MOPS) pH 7.0 and diluting with 50 mm MOPS pH 7.0 immediately before use. H_2_O_2_ stock solutions were prepared fresh, and the concentration was verified by measuring the absorbance at 240 nm using an extinction coefficient of 43.6 cm^−1^·m ^−1^.

### Enzyme expression and purification

The chitinolytic AA10 LPMO from *Serratia marcescens* (*Sm*AA10A; also known as CBP21) was expressed and purified as previously described using hydrophobic interaction chromatography with a chitin bead column [8].

The fungal LPMO from *Neurospora crassa* (*Nc*AA9C) was expressed and purified as previously described using a combination of hydrophobic interaction and anion‐exchange chromatography [39, 53].

### Copper saturation

To ensure copper saturation, the purified enzymes were incubated with a 3‐fold molar excess of CuSO_4_ for 30–60 min at 4 °C in 50 mm Tris(hydroxylmethyl)aminomethane hydrochloride (Tris/HCl) pH 8.0, followed by removal of excess free copper. Two methods for removal of free copper were used. For *Sm*AA10A, a series of concentration and dilution steps using 50 mm Tris/HCl, pH 8.0, were performed using the 10 kDa Amicon® Ultra‐15 centrifugal filter unit (Merck, Darmstadt, Germany) to reach a minimum dilution factor of 1 000 000‐fold. For *Nc*AA9C, a HiPrep 26/10 desalting column was used to remove the excess free copper and change the buffer to 50 mm Tris/HCl, pH 7.0, and 100 mm NaCl. In both cases, the enzyme preparations were controlled for excess free copper by monitoring the (absence of) copper‐mediated oxidation of ascorbate in an ultrafiltrate of the final enzyme preparation, using the Amplex Red/horseradish peroxidase reaction as previously described [39, 41].

### LPMO oxidase activity

The oxidase activity was measured using a protocol adapted from [39, 54]. In brief, 90 μL of a reaction mixture containing 50 mm MOPS, pH 7.0, 0.1 mm Amplex Red (AR), 5 U·mL^−1^ horse radish peroxidase (HRP) and 1 mm ascorbate was incubated for 5 min at 30 °C before adding 10 μL of enzyme to a final concentration of 1 μm for *Nc*AA9C and 3 μm *Sm*AA10A. The production of H_2_O_2_ was monitored at 542 nm over 20 min, and the initial rate was determined from the linear portion of the product formation curve.

### Chitin degradation by *Sm*AA10A

Reactions with *Sm*AA10A were performed by mixing 1 μm enzyme, 50 mm MOPS, pH 7.0, and β‐chitin (0.5–10 g·L^−1^) for a 30 min pre‐incubation at 30 °C with 850 rpm agitation in a ThermoMixer Comfort (Thermo Fisher Scientific, Waltham, MA, USA). The reactions were then initiated by adding ascorbate to a final concentration of 25–1000 μm. In reactions with exogenously added H_2_O_2_, the H_2_O_2_ was added to a final concentration of 100 μm immediately before the addition of ascorbate. For reactions containing cyanide, cyanide from a concentrated stock solution was added at the 15‐min mark during the pre‐incubation of the enzyme with the substrate.

The reactions were terminated at different time points by filtering 75 μL aliquots through a 0.45 μm MultiScreen™ 96‐well filter plate (Merck). The filtrates were incubated with 1 μm chitobiase (*Sm*CHB) for 16 h at 37 °C to degrade the solubilized oxidized chitooligomers to the oxidized dimer (chitobionic acid, GlcNAcGlcNAc1A) and the native monomer (GlcNAc), which simplifies analysis and product quantification.

To determine the total amount of oxidized products, 75 μL sample aliquots were transferred to microtubes, followed by enzyme inactivation through boiling for 10 min. The samples were cooled and then diluted two‐fold before adding 1 μm Chitinase A from *Serratia marcescens* (*Sm*ChiA) and 1 μm *Sm*CHB, followed by incubation at 40 °C for 24 h.

### Substrate degradation by *Nc*AA9C

*Nc*AA9C reactions were performed by incubating 1 μm enzyme, 50 mm MOPS, pH 7.0, and cellopentaose (25–1000 μm) (Megazyme, Bray, Ireland) or 0.1% (w/v) phosphoric acid swollen cellulose (PASC), prepared from Avicel according to [55], for a 30 min pre‐incubation at 30 °C with 850 rpm agitation in a ThermoMixer Comfort (Thermo Fisher Scientific). The reactions were initiated by adding 25–1000 μm ascorbate. In reactions with exogenously added H_2_O_2_, the H_2_O_2_ was added to a final concentration of 100 μm immediately before adding ascorbate. For reactions containing cyanide, cyanide from a concentrated stock was added at the 15‐min mark during the pre‐incubation of enzyme with the substrate.

For reactions performed with cellopentaose, 25 μL samples were taken at varying timepoints, which were diluted 10‐fold in 200 mm NaOH, to terminate the reaction, followed by filtering through a 0.45 μm MultiScreen™ 96‐well filter plate (Merck).

Reactions with PASC were terminated by sampling 65 μL at various time points, followed by boiling for 10 min, and filtering through a 0.45 μm MultiScreen™ 96‐well filter plate (Merck).

### HPLC analysis of chitin‐derived products

Analysis of chitobionic acid and the native monomer was performed using an Ultimate 3000 RSLC UHPLC (Dionex, Sunnyvale, CA, USA) by injecting an 8 μL sample onto a 100 × 7.8 Rezex RFQ‐Fast acid H+ (8%) column (Phenomenex, Torrance, CA, USA) operated at 85 °C with a flow rate of 1 mL·min^−1^. An isocratic gradient of 5 mm sulfuric acid was used. Products were detected by UV at 194 nm and chitobionic acid was quantified using in‐house generated standards (25–1600 μm) as previously described [12, 56].

Since ascorbate co‐elutes with chitobionic acid, an alternative analytical method was used for experiments involving various concentrations of ascorbate, involving an Agilent 1290 Infinity II UHPLC (Agilent, Santa Clara, CA, USA) equipped with a 2.1 × 50 mm, 130 Å, 1.7 μm BEH Amide column (Waters, Milford, MA, USA) and a 5 mm VanGuard pre‐column with the same column material. The analysis was performed by injecting a 5 μL sample with a flow rate of 0.4 mL·min^−1^ at 25 °C followed by isocratic elution with 78% acetonitrile and 22% 15 mm Tris/HCl, pH 8.0. All samples were adjusted to 78% (v/v) acetonitrile before injection.

### HPLC analysis of cellulose‐derived products

Products generated from PASC or cellopentaose were analyzed using High Performance Anion Exchange Chromatography with Pulsed Amperometric Detection (HPAEC‐PAD) on an ICS 6000 (Dionex) equipped with a 1 × 250 mm CarboPac PA200 (Thermo Fisher Scientific) column and a 1 × 50 mm guard column of the same material, as described before [57]. The flow rate was 62 μL · min^−1^, and the column oven temperature was 30 °C.

For quantification of the native trimer, a 27‐min method was used with a dual eluent generator creating the KOH and potassium methanesulfonate (KMSA) eluents, with the following gradient: 0–30 mm KMSA over 0–6 min; 30–100 mm KMSA over 4 min and held for an additional 5 min; 100–0 mm KMSA over 0.1 min; 0 mm KMSA held for 11.9 min. The KOH concentration remained fixed at 100 mm throughout the run. Quantification of the native trimer was performed using purchased cellotriose as a standard (Megazyme).

For quantification of C4‐oxidized products, a 26‐min method was used with the following gradient: 1–100 mm KMSA over 14 min and held for an additional 3 min; 100–1 mm KMSA over 0.1 min; 1 mm KMSA for 8.9 min. The KOH concentration remained fixed at 100 mm throughout the run. Quantification of the C4‐oxidized dimer and trimer was performed using in‐house generated standards as previously described [57].

### Transient state stopped‐flow kinetics

The transition between LPMO‐Cu(II) and LPMO‐Cu(I) in both the presence and absence of cyanide was analyzed using a SFM 4000 stopped‐flow equipped with a MOS 200 M dual absorbance spectrofluorometer (BioLogic, Seyssinet‐Pariset, France). To ensure single turnover conditions, all reactions were performed anaerobically by storing N_2_ purged buffer and labware in an A95TG anaerobic workstation (Don Whitley, West Yorkshire, UK) for 16 h before usage. Enzyme, ascorbate, and cyanide solutions were prepared fresh in the chamber and sealed in syringes before transferring to the stopped‐flow syringe handling unit, which had been flushed with N_2_ purged buffer. For determining reduction and oxidation rates, the fluorescence shift between LPMO‐Cu(II) and LPMO‐Cu(I) [58] was monitored using a PMT‐250 photomultiplier tube (BioLogic) with a set voltage of 600 V.

For reactions without cyanide, the reduction was assessed by a single mixing experiment, mixing 5 μm LPMO‐Cu(II) with increasing concentrations of ascorbate (10–200 μm) in 50 mm MOPS, sodium phosphate, Tris/HCl or 2‐[Bis‐(2‐hydroxyethyl)‐amino]‐2‐hydroxymethyl‐propane‐1,3‐diol (Bis‐Tris), pH 7.0. In reactions with cyanide and when measuring LPMO oxidation, we used a double mixing setup with a 100 μL delay line. For reduction in the presence of cyanide, 5 μm LPMO‐Cu(II) was mixed with an equal volume of solutions with increasing concentrations of cyanide (10–2000 μm) and stored in the delay line for 10 s, followed by a second mixing with 200 μm ascorbate.

For reoxidation experiments, 10 μm LPMO‐Cu(II) was mixed with equimolar amounts of L‐cysteine and stored for 10 s in the delay line to ensure reduction before mixing with increasing concentrations of H_2_O_2_ (10–800 μm). These experiments were performed in 50 mm MOPS, pH 7.0.

For all cases, the pseudo‐first order reaction rates (*k*_obs_) were determined by solving a baseline corrected single exponential equation ($y=\mathrm{at}+b+ce^{-k_{\mathrm{obs}}t}$).

### Monitoring of H_2_O_2_ consumption with an electrochemical sensor

To monitor the real‐time consumption of H_2_O_2_, a Prussian blue‐coated rotating disk gold electrode was used as described in [27]. In short, the electrochemical setup consisted of a rotating disk gold working electrode, a double junctioned Ag|AgCl reference electrode with a 3 m KCl supporting electrolyte and an in‐house customized platinum sheet counter electrode. To selectively react with H_2_O_2_, the gold working electrode was modified by electro‐depositing a thin layer of Prussian blue, which was achieved by performing 6–8 cycles of staircase cyclic voltammetry, applying a potential scan between 600 and 900 mV vs standard hydrogen electrode (SHE) in a solution of 0.1 m KCl, 1 mm K_3_[Fe(CN)_6_], 1 mm FeCl_3_ and 0.1 m HCl. After the Prussian blue deposition, the working electrode was activated in 0.1 m KCl and 0.1 m HCl by performing 20 cyclic voltammetry staircase scans between 160 and 590 mV vs SHE. Only working electrodes obtaining anodic peak densities between 2 and 4 nA·cm^−2^ during activation were used. After activation, the gold electrode was covered with a layer of Nafion™ (5% in aliphatic alcohols, Merck) by pipetting 7 μL undiluted Nafion onto the electrode surface and cured overnight to protect the Prussian blue coating from abrasion during later use. Before analysis, the working electrode was activated in the buffer used for analysis with 0.1 m KCl by performing 20 CV staircase scans between 160 and 590 mV vs SHE.

Reactions with *Nc*AA9C were performed with 4 g·L^−1^ tamarind seed xyloglucan (Megazyme) as substrate, while reactions with *Sm*AA10A were performed with 10 g·L^−1^ β‐chitin. Both reactions were prepared in 0.1 m KCl and 50 mm buffer, pH 7.0 (MOPS, sodium phosphate, Tris/HCl, or Bis‐Tris). Real‐time monitoring of H_2_O_2_ consumption was performed using chronoamperometry by applying a potential of 100 mV vs SHE. A typical reaction was performed using an angular velocity of 50 s^−1^ and was preceded by an electrode polarization step during which the signal was monitored for 45–60 s to obtain a stable signal. An internal calibration of each run was performed by five consecutive additions of known amounts of H_2_O_2_ before initiating the LPMO reaction, reaching a total starting concentration of H_2_O_2_ of either 50 μm (*Sm*AA10A) or 100 μm (*Nc*AA9C). After this calibration step, the enzyme was added and mixed for approx. 30 s before adding ascorbate (100 μm) to start the LPMO reaction.

### H_2_O_2_ sensor data analysis

With electrochemical detection of H_2_O_2_ using a rotating disk electrode, the reduction in the H_2_O_2_ concentration over time is observed as a change in current over time, as thoroughly described in [27]. The raw traces were first corrected for a system drift, which was performed using a two‐point linear baseline correction. One point was selected during the electrode polarization (pre‐experiment), and the second in the post‐experimental baseline. In reactions that terminated prior to consuming all the H_2_O_2_, the system drift was determined in the post‐experimental baseline over a duration of 30–60 s. Following the baseline correction, linear regression was performed on the currents measured during the five‐step calibration procedure, yielding the relationship between current, in nano ampere (nA), and the concentration of H_2_O_2_ in μm. Following the conversion from current to μm H_2_O_2_, figures were prepared showing only the data points from the reaction start until the reaction, meaning that the figures only show LPMO‐related H_2_O_2_ consumption. All data analysis can be performed using a command line interface, which is available on GitHub (https://github.com/ogo001/H2O2_RDE). A schematic representation of the data analysis is presented in Fig. S1. [27].

### Reactions with 2,6‐dimethoxyphenol (2,6‐DMP)

In the presence of H_2_O_2_, LPMOs oxidize the chromogen 2,6‐DMP in what essentially is a peroxidase reaction, resulting in the reduction of H_2_O_2_ to water and the formation of the colored compound coerulignone that absorbs at 469 nm [59]. To assess inhibition of this peroxidase activity by cyanide or sodium phosphate, reaction mixtures were assembled consisting of 2 μm *Sm*AA10A or *Nc*AA9C in 50 mm MOPS, pH 7.0, with 1 mm 2,6‐DMP and 50 μm H_2_O_2_. Reactions were performed at 30 °C in triplicate in 50 μL volumes in a 96‐well plate. Cyanide (0–1000 μm) or sodium phosphate pH 7.0, (0–500 mm) were added to the reaction mixture from concentrated stock solutions. Reactions were initiated by adding 2 μL of concentrated LPMO solution to 48 μL of a pre‐mixed, pre‐warmed solution of MOPS, 2,6‐DMP, H_2_O_2_ and inhibitor (cyanide or sodium phosphate), followed by brief mixing. Absorbance was monitored at 473 nm using a Multiskan™ FC microplate photometer (Thermo Fisher Scientific). Measurements were recorded every 1 s for 2 min, starting 10 s after the reactions were initiated. The reported values reflect the initial, linear rate of the increase in absorbance recorded between 10 and 20 s after the initiation of the reaction.

### Electron paramagnetic resonance (EPR)

Continuous‐wave X‐band (~ 9.47 GHz) EPR spectra were collected on a Bruker Magnettech ESR5000 (Bruker, Billerica, MA, USA) using custom‐made quartz EPR tubes with an outer diameter of 4 mm. Samples were frozen in liquid nitrogen (77 K). Data was collected at 100 K using a sweep time of 60 s, modulation frequency of 100 kHz, modulation amplitude of 1 mT and microwave power of 10 mW. All spectra were analyzed in Matlab using the EasySpin 6.0.0 package [60].

### Detection of hydroxyl radicals

The specific radical trap terephthalic acid will react with OH^•^ to generate 2‐hydroxyterephthalic acid which is fluorescent. Using a Varioskan Lux (Thermo Fisher Scientific) in fluorescence mode, the formation of OH^•^ in Fenton‐like reactions in various buffers was monitored. The reactions contained 5 μm CuSO_4_, 1 mm ascorbate, 100 μm H_2_O_2_, 100 μm terephthalic acid and 50 mm buffer pH 7.0 (MOPS, Bis‐Tris, Tris/HCl or sodium phosphate) and radical formation was monitored for 30 min at 30 °C, using a 312 nm excitation and a 328 nm emission wavelength.

## Results

### Comparison of SmAA10A and NcAA9C

*Sm*AA10A, a chitin‐active bacterial LPMO [7, 8], and *Nc*AA9C, a fungal LPMO active on cellulose, cello‐oligomers and several hemi‐cellulosic glycans [39, 61, 62] are among the best studied LPMOs. The *Sm*AA10A active site displays a trigonal‐bipyramidal copper configuration with phenylalanine (*Sm*AA10A; Phe187) in the axial position, whilst *Nc*AA9C presents a tetragonal active site with a tyrosine (*Nc*AA9C; Tyr166) in the axial position (Fig. 2A). These structural features are reflected in altered copper electronics yielding a more rhombic and a more axial EPR spectrum for *Sm*AA10A and *Nc*AA9C, respectively [63, 64]. Expanding beyond the first coordination sphere, the less conserved second sphere residues partake in modulating copper reactivity but do not directly interact with the copper atom. The importance of second sphere residues for catalysis has been demonstrated in several studies using different experimental approaches [21], including studies of *Sm*AA10A [8, 18] and *Nc*AA9C [19]. Here, we have assessed the differences between these two enzymes in more detail, using identical methods and experimental conditions for both.

Oxidase activity, i.e., LPMO‐catalyzed oxidation of a reductant leading to formation of H_2_O_2_, was monitored using the Amplex Red/HRP method [39, 54] in 50 mm MOPS, pH 7.0, and revealed an almost 10‐fold difference in rate (Fig. 2B, 0.002 and 0.015 s^−1^ for *Sm*AA10A and *Nc*AA9C, respectively). These differences were further analyzed using transient state stopped‐flow kinetics to probe the reduction rates with ascorbate (Fig. 2C) and oxidation rates with H_2_O_2_ (Fig. 2D). *In situ* mixing of LPMO‐Cu(II) with increasing ascorbate concentrations performed under anaerobic pseudo‐first‐order conditions in 50 mm MOPS, pH 7.0, yielded reduction rates of 527 000 ± 15 000 m ^−1^·s^−1^ for *Sm*AA10A and 167 000 ± 5700 m ^−1^·s^−1^ for *Nc*AA9C (Fig. 2C). Oxidation of *Sm*AA10A and *Nc*AA9C by H_2_O_2_ happened with rates amounting to 7500 ± 300 m ^−1^·s^−1^ and 72 600 ± 2000 m ^−1^·s^−1^, respectively (Fig. 2D). Compared to *Nc*AA9C, *Sm*AA10A has a higher reduction rate and a lower LPMO oxidation rate with both O_2_ (oxidase assay) and H_2_O_2_ (stopped‐flow data), which aligns with *Sm*AA10A having a more positive reduction potential [275 ± 6 mV vs SHE [65], pH 7.0, vs 211 ± 2 mV vs SHE for *Nc*AA9C [19], pH 6.5]. The two enzymes showed similar activities in the peroxidase assay with 2,6‐DMP (Fig. 2E). The rate of formation of the final product in this reaction depends on two rates, the rate of LPMO reduction by 2,6‐DMP and the rate of LPMO oxidation by H_2_O_2_ [59]. The rate of reduction is higher for *Sm*AA10A (when measured with ascorbate) whilst the rate of oxidation by H_2_O_2_ is higher for *Nc*AA9C, which may explain why the two enzymes show similar overall rates.

The effects of cyanide on LPMO reduction rates were then studied to gain more insight into the reduction kinetics.

Strikingly, the reduction of *Nc*AA9C was severely inhibited by cyanide, showing almost full inhibition when using equimolar amounts of cyanide (Fig. 2F, yellow squares). Reduction of *Sm*AA10A was also clearly inhibited by cyanide, but inhibition was less severe as 2 mm cyanide did not fully inhibit *Sm*AA10A (Fig. 2F, blue bullets). The results reveal a difference between the two LPMOs and suggest that cyanide binds to Cu(II), thus competing with the reductant and preventing reduction. Control reactions showed that exposure to cyanide does not affect the fluorescence signal used to monitor LPMO reduction (Fig. S2). Another control experiment without a reductant showed that cyanide cannot drive the LPMO reaction (Fig. S3). Both control experiments also show that cyanide is unable to reduce the LPMO.

### EPR spectroscopy

**Fig. 3:**
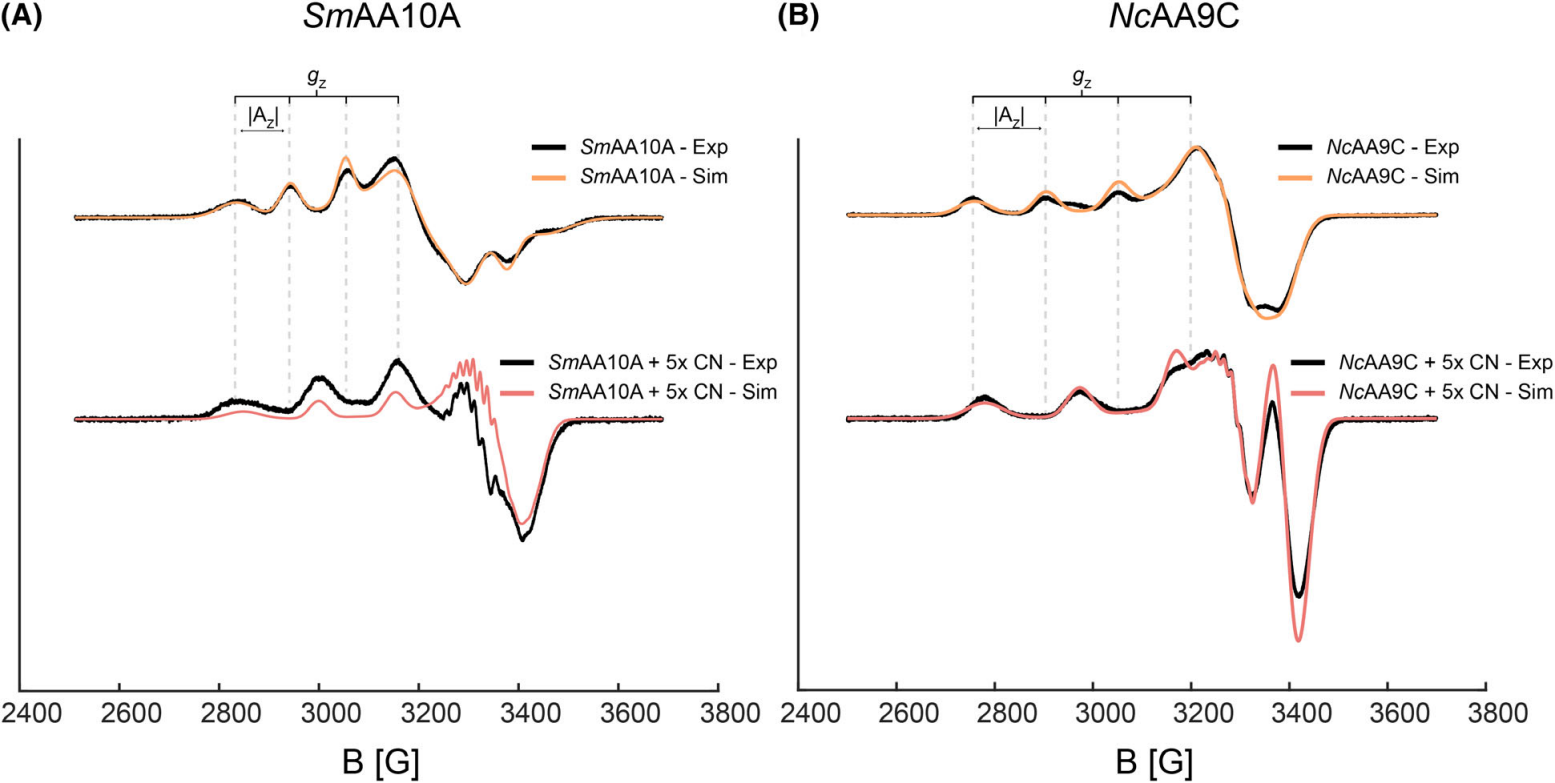
CW X‐band EPR spectroscopy. The spectra were collected on 400 μm *Sm*AA10A (A) or *Nc*AA9C (B), both in the absence and presence of 2 mm potassium cyanide. All samples were prepared in 50 mm MOPS, pH 7.0, and frozen in liquid nitrogen before collecting the spectra at 100 K. Data were collected using a sweep time of 60 s, modulation frequency of 100 kHz, modulation amplitude of 1 mT and microwave power of 10 mW. The raw data traces are black, and the simulations performed using the Easy Spin 6.0.0 Matlab package are presented in colors. Spin Hamiltonian parameters are shown in Table S1.

EPR spectroscopy is well suited for the Cu(II) active site due to the d^9^ electronic configuration yielding a paramagnetic system, whereas the Cu(I) d^10^ electronic configuration is EPR silent. Using a continuous‐wave (CW) X‐band EPR spectrometer, the spectra of the LPMOs, alone or with a five‐fold molar excess of cyanide, were collected (Fig. 3).

The *Sm*AA10A EPR spectrum in the absence of cyanide is rhombic (*g*_x_ ≠ *g*_y_ ≠ *g*_z_), in line with the trigonal‐bipyramidal active site configuration observed for chitin‐active AA10 LPMOs with an axial phenylalanine [66, 67]. The successful simulation of the *Sm*AA10A spectra using rhombic *g* tensors further supports the presence of a trigonal‐bipyramidal active site (Fig. 3A, Hamiltonian parameters given in Table S1). The EPR spectrum of *Sm*AA10A with cyanide show more axial *g* tensors (Fig. 3A). Compared to the spectrum without cyanide, the *A*_||_ increases from 355 to 472 MHz, and the nitrogen superhyperfines become visible. This change in active site electronic geometry clearly indicates the influence of cyanide on Cu(II) and may indicate a transition towards a square planar active site.

As expected, considering the tetragonal conformation of the copper site and results of previous studies [64], *Nc*AA9C has an axial EPR spectrum (*g*_x_ = *g*_y_ ≠ *g*_z_) in the absence of cyanide (Fig. 3B). The addition of cyanide has effects on the copper similar to those seen for *Sm*AA10A. Cyanide increases the *A*_||_ from 465 to 600 MHz, and the nitrogen superhyperfines become resolved.

The addition of cyanide to both enzymes resulted in the *m*_I_ = −3/2 energy transition becoming visible, in line with the increased ^63/65^Cu hyperfine distance (*A*_||_). Simulations with and without cyanide yielded confident *g*_||_ and *A*_||_ values in the parallel region, however, lack of resolution in the perpendicular region excluded confident simulation of *A*_x,y_ and *g*_x,y_ values.

### The effect of cyanide on substrate oxidation

The results reported above show that cyanide interacts with the active site copper of both LPMOs and that this interaction is stronger for *Nc*AA9C compared to *Sm*AA10A. Monitoring LPMO reactivity without substrate addresses off‐pathway reactions that provide insight into LPMO copper reactivity. However, the physiologically relevant reaction occurs in the presence of a polysaccharide substrate. To investigate the effect of cyanide on catalysis, cyanide was added to reactions that were either *in situ* H_2_O_2_‐limiting or that were supplied with externally added H_2_O_2_. Importantly, the time scales of these reactions are very different; for most of the reactions with externally added H_2_O_2_ reported below, the reaction was complete at the first of three reported measuring time points (i.e., after 3 min), whereas progress curves spanning multiple hours were obtained when using *in situ* H_2_O_2_‐limiting conditions.

**Fig. 4:**
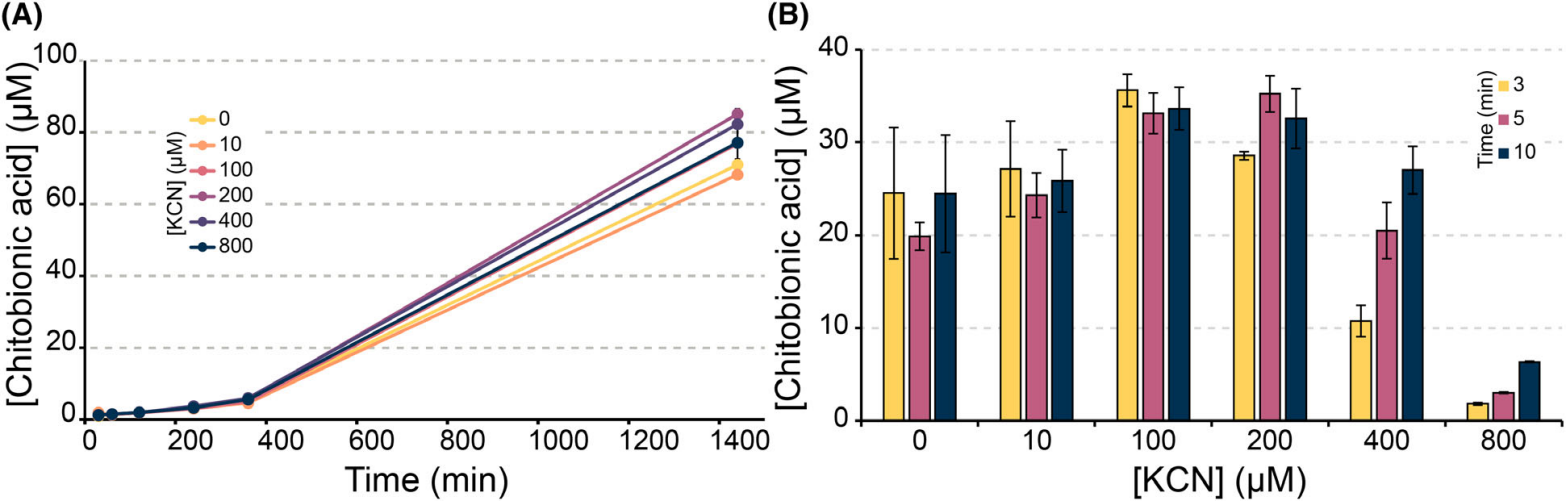
Cyanide inhibition of chitin degradation by *Sm*AA10A. (A) *Sm*AA10A reactions were performed with 1 μm enzyme, 10 g∙L^−1^ β‐chitin and cyanide (0–800 μm) in 50 mm MOPS, pH 7.0, and initiated by adding 1 mm ascorbate (*in situ* H_2_O_2_‐limiting conditions). (B) *Sm*AA10A reactions were performed with 1 μm enzyme, 10 g∙L^−1^ β‐chitin, cyanide (0–800 μm), and 100 μm exogenously added H_2_O_2_ in 50 mm MOPS, pH 7.0, and initiated by adding 100 μm ascorbate. For both reaction setups, soluble products were converted to native monomer (GlcNAc) and oxidized dimer (GlcNAcGlcNAc1A) and the latter was quantified. All reactions were performed in triplicates, standard deviations are shown as error bars.

Reactions of *Sm*AA10A with β‐chitin under *in situ* H_2_O_2_‐limiting conditions showed a slow release of oxidized products (Fig. 4A), which correlates with the low oxidase activity of this enzyme (Fig. 2B). Cyanide did not inhibit this reaction, which may seem strange in light of the impact of cyanide on the reduction of *Sm*AA10A (Fig. 2F). However, under these conditions, substrate turnover is limited by *in situ* generation of H_2_O_2_ and, thus, is very slow (in the order of 0.002 s^−1^), which means that the reduced reduction rate will not become rate‐limiting. It is also worth noting that, considering the much higher rate of the peroxygenase reaction (in the order of 1–10 s^−1^; see below and [68]), only a minor fraction of reduced LPMO is needed to ensure immediate productive use of emerging H_2_O_2_. Figure 4A shows that cyanide caused a slight increase in product formation after 24 h; similar minor boosting effects can be observed in several of the experiments shown hereafter and are discussed further below.

In contrast, in reactions driven by exogenously added H_2_O_2_, cyanide inhibition became clearly visible at cyanide concentrations of 200 μm and higher (Fig. 4B). In these reactions, the reductant concentration is lower (100 μm), allowing the cyanide to better compete with ascorbate and reduce the reduction rate. Furthermore, in these fast reactions, with a surplus of H_2_O_2_, the concentration of active enzyme (i.e., reduced enzyme) will affect product formation, explaining why inhibition of reduction, in this case, translates into reduced product formation. Control reactions were performed to exclude an interaction between H_2_O_2_ and cyanide, showing that the concentration of H_2_O_2_ remains stable in the presence of cyanide (Fig. S4).

**Fig. 5:**
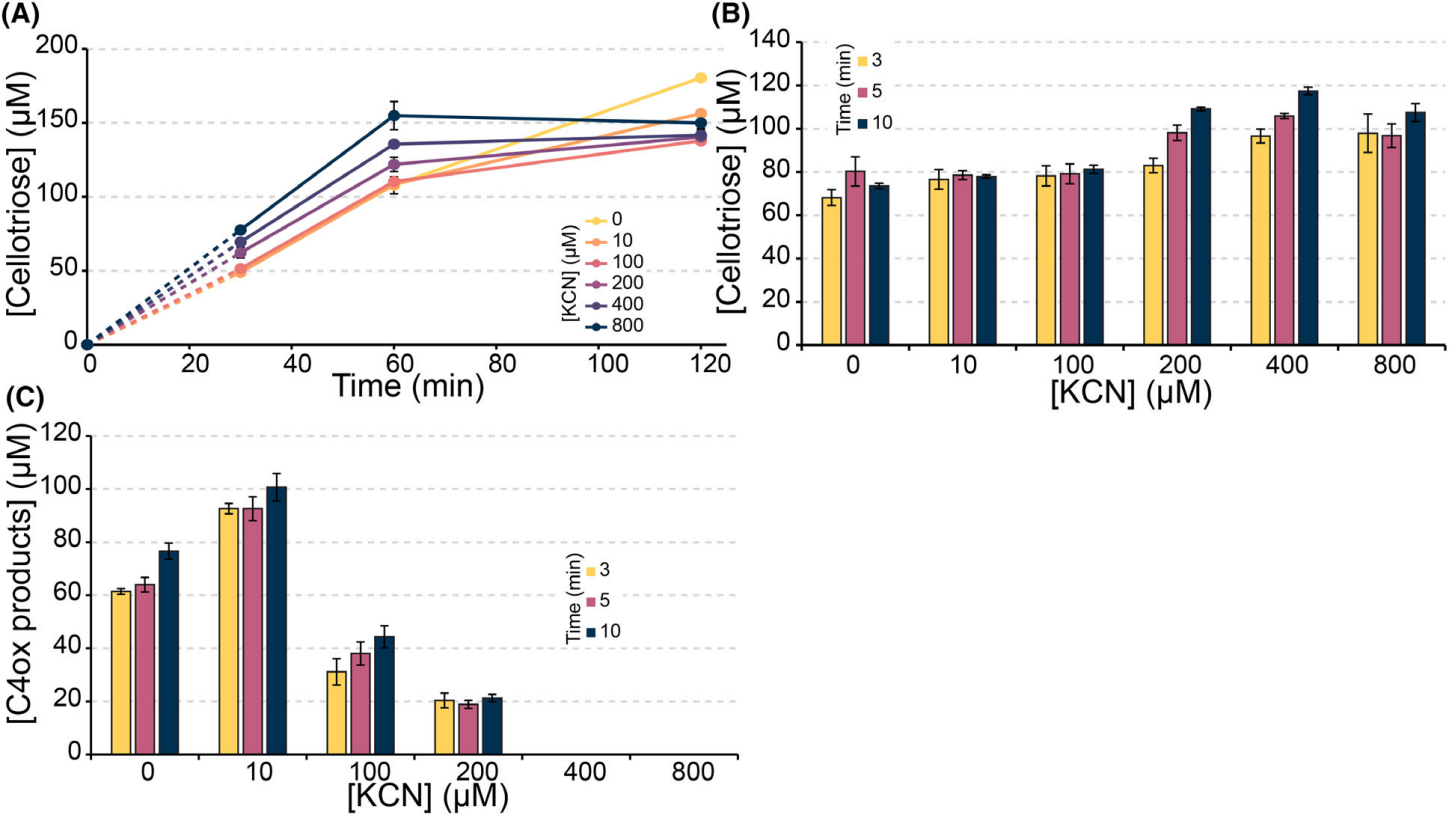
Cyanide inhibition of the degradation of cellopentaose and PASC by *Nc*AA9C. (A) *Nc*AA9C reactions were performed with 1 μm enzyme, 1 mm cellopentaose and cyanide (0–800 μm) in 50 mm MOPS, pH 7.0, and initiated by adding 1 mm ascorbate. (B) *Nc*AA9C peroxygenase reactions were performed with 1 μm enzyme, 1 mm cellopentaose, 100 μm exogenously added H_2_O_2_, and 0–800 μm cyanide in 50 mm MOPS, pH 7.0, and initiated by adding 100 μm ascorbate. (C) *Nc*AA9C reactions were performed with the same reaction conditions as in B; however, instead of 1 mm cellopentaose, 0.9% (w/v) PASC was used. For reactions performed using cellopentaose (A and B), cellotriose was quantified, whilst for reactions performed using PASC (C), the C4 oxidized dimer and trimer were quantified as described previously [57]. Standard deviations are shown as error bars (*n* = 3) for all reactions.

Similar to what was observed for *Sm*AA10A, and despite more efficient inhibition of reduction by cyanide in the absence of substrate (Fig. 2F), reactions with *Nc*AA9C acting on cellopentaose under *in situ* H_2_O_2_‐limiting conditions did not show inhibition of substrate conversion by cyanide. This underpins that reduction is not limiting the reaction under these conditions, as discussed above. Similar to the reactions with *Sm*AA10A, the addition of cyanide, led to slightly increased product formation (Fig. 5A), which, in this case was accompanied by potential signs of enzyme inactivation (i.e., progress curves level off). These unexpected cyanide effects are addressed further in the Discussion section. To simplify interpretation of data, reaction setups with exogenously added H_2_O_2_ were used in subsequent experiments. Under these conditions, the oxidase activity will not be favored and, besides, the time frame of these reactions is so short that the effects of the slow oxidase reactions (and slow abiotic oxidation of the reductant) are expected to be small.

Interestingly, cyanide inhibition did not occur for *Nc*AA9C acting on cellopentaose in reactions with 100 μm of exogenously added H_2_O_2_ (Fig. 5B), contrasting the results obtained with *Sm*AA10A acting on chitin (Fig. 4B). It has been shown previously that conversion of small, soluble and easily diffusible cellopentaose by *Nc*AA9C in reactions with added H_2_O_2_ is extremely efficient [27, 69] and it is plausible that substrate interaction kinetics for this substrate are very different compared to an insoluble substrate. Efficient substrate binding will reduce off‐pathway reoxidation of the enzyme through the peroxidase reaction, which will reduce the need for re‐reduction as well as the impact of the inhibitory effect of cyanide on this reduction. Indeed, when using the insoluble substrate PASC, clear inhibition by cyanide did occur and full inhibition was observed at the higher concentrations (400–800 μm) (Fig. 5C). Notably, inhibition of *Nc*AA9C acting on PASC happened at lower concentrations of cyanide compared to *Sm*AA10A acting on chitin (Fig. 4B), in line with the stronger effect of cyanide on reduction of *Nc*AA9C (Fig. 2F). Again, a small boosting effect of cyanide was observed at the lowest tested cyanide concentration (10 μm), which, notably, is some 10‐fold lower than concentrations needed to slightly boost activity of *Sm*AA10A. The observed inhibition shows that when using an insoluble, slow diffusing substrate, (re‐)reduction of *Nc*AA9C becomes a limiting factor and cyanide effects on reduction become noticeable. Taken together, the results so far show that the impact of cyanide on LPMO activity is both LPMO‐ and substrate‐dependent.

### The impact of reductant and substrate concentrations on LPMO inhibition

**Fig. 6:**
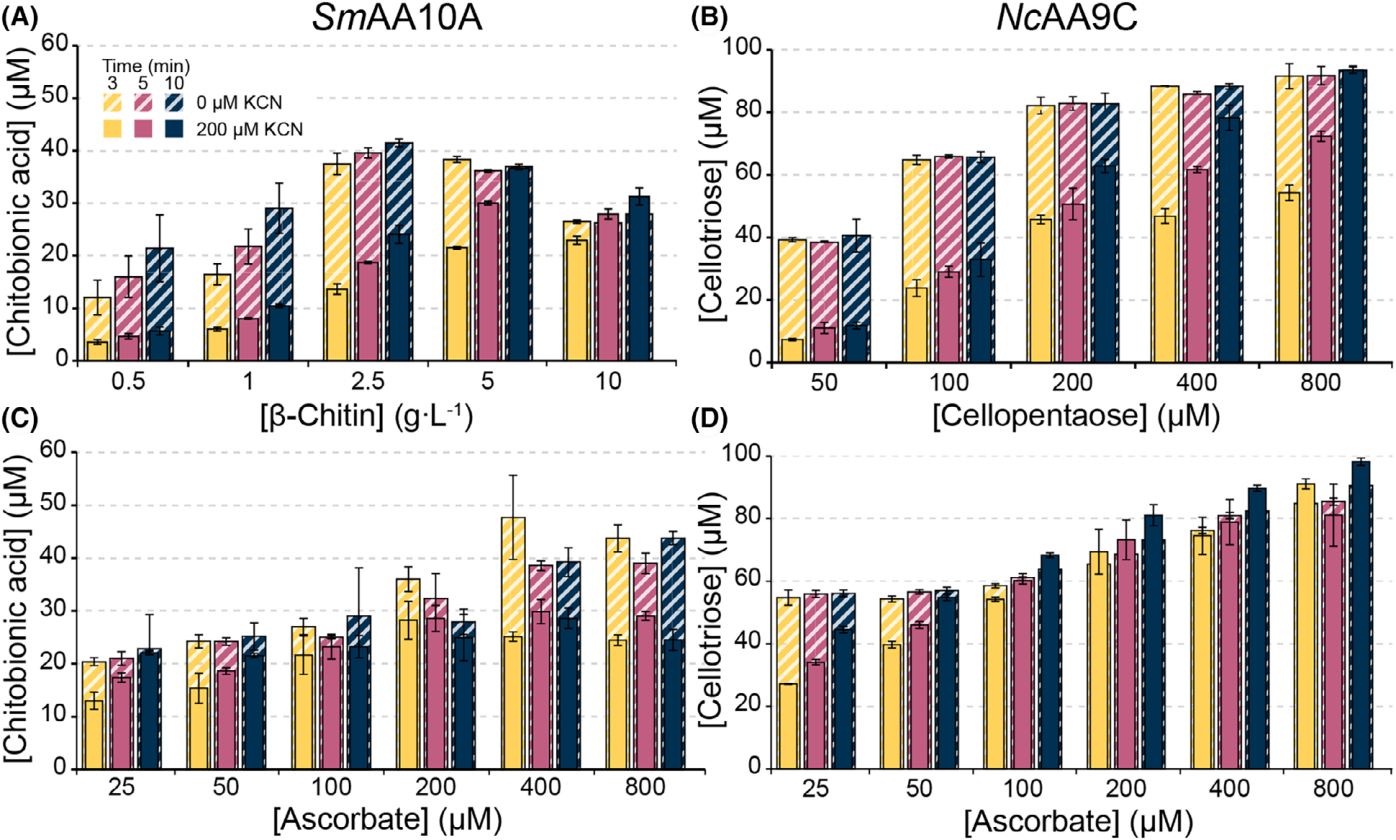
The effect of substrate and reductant concentration on LPMO reactions. Substrate degradation was performed with 1 μm enzyme in 50 mm MOPS, pH 7.0, and 100 μm added H_2_O_2_, at 30 °C with 850 rpm agitation, varying either substrate or reductant concentration. Reactions were started by adding 100 μm ascorbate. For all reactions, time points were taken at 3, 5 and 10 min, and reactions were performed in the absence (striped bars) or presence of 200 μm cyanide (full‐shaded bars). Panels A, C: *Sm*AA10A reactions performed with (A) varying β‐chitin concentrations (0.5–10 g·L^−1^) with a fixed ascorbate concentration (100 μm) and (C) varying ascorbate concentrations (25–800 μm) with a fixed concentration of β‐chitin (10 g·L^−1^). Panels B, D: *Nc*AA9C reactions with (B) varying cellopentaose concentrations (50–800 μm) with a fixed ascorbate concentration (100 μm) and (D) varying ascorbate concentrations (25–800 μm) with a fixed concentration of cellopentaose (1 mm). For reactions performed with *Nc*AA9C, the cellotriose product was quantified using a Dionex ICS 6000 HPLC and for reactions performed with *Sm*AA10A oxidized oligomers were degraded to the oxidized dimer (chitobionic acid), which was quantified using either a Dionex Ultimate 3000 UHPLC (A) or an Agilent 1290 UHPLC (C). All reactions were performed in triplicates, and standard deviations are shown as error bars for *n* = 3.

To further assess substrate and reductant effects in the inhibition of the LPMO reaction by cyanide, the concentrations of these components were varied in reactions with 0 or 200 μm cyanide. For *Nc*AA9C, we focused on the reaction with cellopentaose, because the lack of cyanide inhibition of *Nc*AA9C in reactions with 1 mm cellopentaose (Fig. 5A,B), contrasting with significant inhibition in reactions with PASC (Fig. 5C), warranted further investigation. Interestingly cyanide inhibition became detectable and increasingly prominent at lower cellopentaose concentrations (Fig. 6B). Likewise, inhibition by cyanide became visible at reductant concentrations below 100 μm, showing that reduction, and, thus, inhibition of reduction, became limiting (Fig. 6D).

Largely similar trends were observed for *Sm*AA10A. Decreasing the concentration of the polymeric substrate β‐chitin led to clearly increased inhibition by cyanide (Fig. 6A). Decreasing the reductant concentration in *Sm*AA10A reactions had a more modest effect on cyanide inhibition compared to *Nc*AA9C (Fig. 6C; compared with Fig. 6D), which aligns well with the lower impact of cyanide on the reduction rate (Fig. 2F). All in all, these results show that the inhibition of both *Nc*AA9C and *Sm*AA10A by cyanide depends on the substrate and the reductant concentration. Cyanide inhibition, caused by the interaction between cyanide and the oxidized LPMO [Cu(II) state], becomes more apparent as reductive activation becomes limiting in the LPMO reaction. This situation emerges at low reductant concentrations, but also at low substrate concentrations, since lack of substrate promotes oxidation of the LPMO through the off‐pathway peroxidase reaction (i.e., futile turnover of H_2_O_2_), meaning that more frequent re‐reduction is needed. The suitability and concentration of the substrate are important determinants of how LPMO reactions proceed, not only in terms of inhibitor sensitivity, but also in terms of enzyme stability, since the peroxidase reaction may damage the enzyme [34, 70].

Enzyme inactivation likely explains the peculiar effects observed when increasing the ascorbate concentration in the reaction with *Sm*AA10A from the standard 100 μm to 400 and 800 μm. In these reactions, product formation increases considerably, and cyanide has a clear inhibitory effect that seems unexpected based on the above observation. We speculate that the increase in activity in the reactions without cyanide is due to the release of free copper by damaged enzymes, which will promote *in situ* generation of H_2_O_2_ through oxidation of ascorbate [41, 42]. Cyanide could inhibit this effect by binding to copper in solution [71]. Previously published data show that enzyme inactivation occurs for *Sm*AA10A under the conditions used here [72] and that such inactivation indeed leads to copper release [42]. Control reactions showed that, indeed, cyanide inhibits LPMO reactions that are driven by copper‐promoted oxidation of ascorbate (Fig. S5).

As an additional control, we also assessed why, for the *Sm*AA10A reactions, the product yields are considerably lower than the 100 μm of product that could be generated when adding 100 μm H_2_O_2_ to the reaction. This low yield of soluble oxidized products (typically 30–40 μm) is expected to be due to the fact that a considerable fraction of LPMO‐generated oxidized sites remain on the insoluble substrate [73]. Control reactions that include analysis of oxidized sites in the insoluble fraction indeed showed higher levels of oxidized products (Fig. S6).

### The impact of buffer ions on LPMO reactivity

Previous work has shown that organic acids and phosphoric acid inhibit the 2,6‐DMP peroxidase reaction catalyzed by *Nc*AA9C [47]. As expected, based on the above observations, cyanide clearly inhibited both *Nc*AA9C and *Sm*AA10A in the 2,6‐DMP assay (Fig. S7), showing that this easy‐to‐use assay is useful for assessing a multitude of inhibitory effects.

**Fig. 7:**
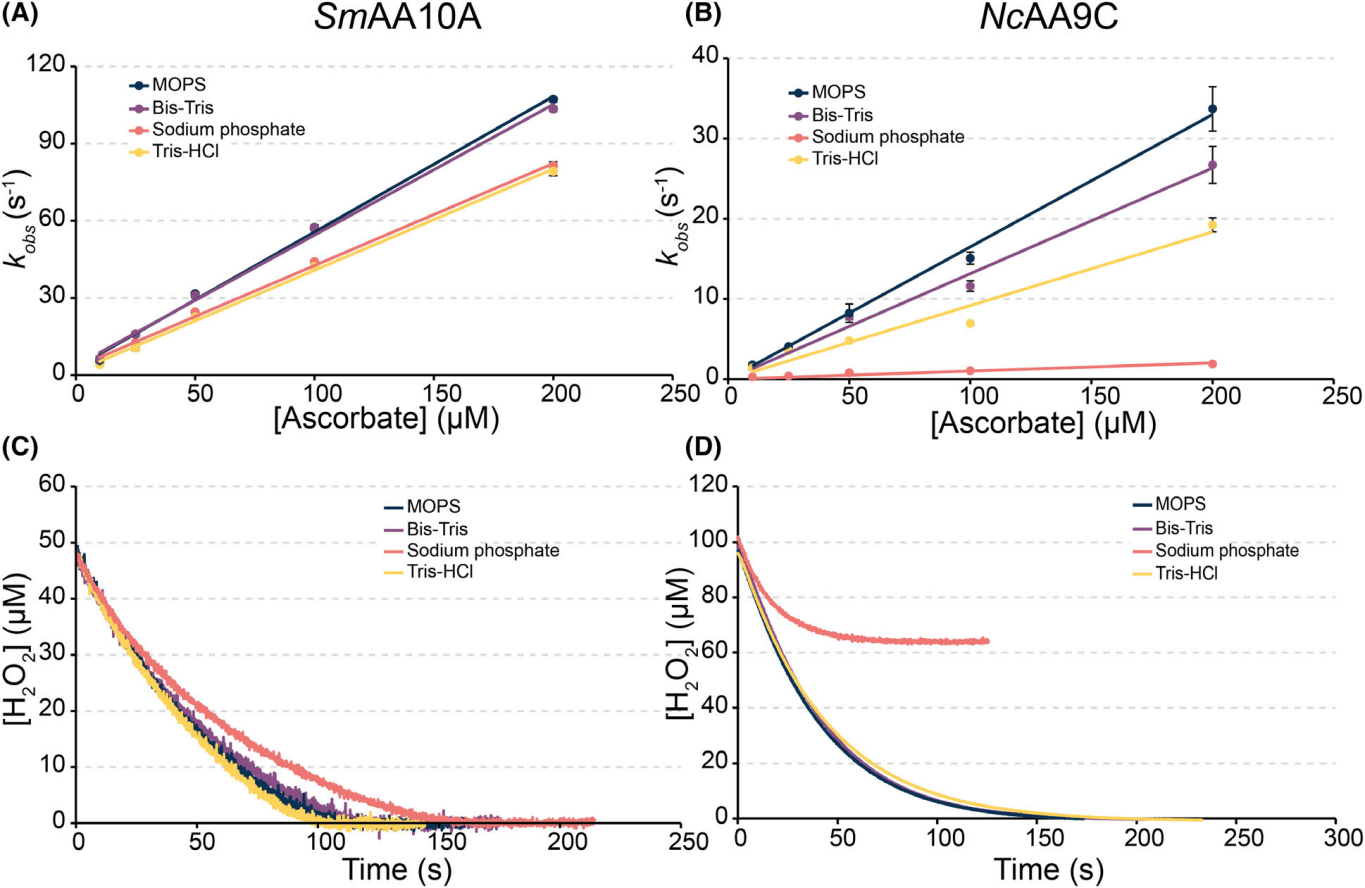
The effect of different buffer molecules on LPMO reactivity. The rate of reduction for (A) *Sm*AA10A and (B) *Nc*AA9C was determined by stopped‐flow mixing of 10 μm enzyme with increasing concentrations of ascorbate (10–200 μm) in 50 mm buffer, pH 7.0. The observed rate (*k*_obs_) was calculated using a single exponential equation $y=\mathrm{at}+b+ce^{-\mathrm{kt}}$ for each ascorbate concentration, and standard deviations are presented as error bars for *n* = 3. (C) Consumption of H_2_O_2_ by 1 μm *Sm*AA10A acting on 10 g·L^−1^ β‐chitin in the presence of 50 μm H_2_O_2_ and after initiation of the reaction by adding 100 μm ascorbate. (D) Consumption of H_2_O_2_ by 50 nm *Nc*AA9C acting on 4 g·L^−1^ xyloglucan (XG) in the presence of 100 μm H_2_O_2_ and after initiation of the reaction by adding 100 μm ascorbate. The reactions depicted in panels C and D were done in 50 mm buffer, pH 7.0, containing 0.1 m KCl in triplicates, and one representative replicate was plotted. Second‐order rate constants derived from panels A and B are listed in Tables S2 and S3. Initial rates derived from panels C and D are shown in Figs S8 and S9, respectively.

**Fig. 8:**
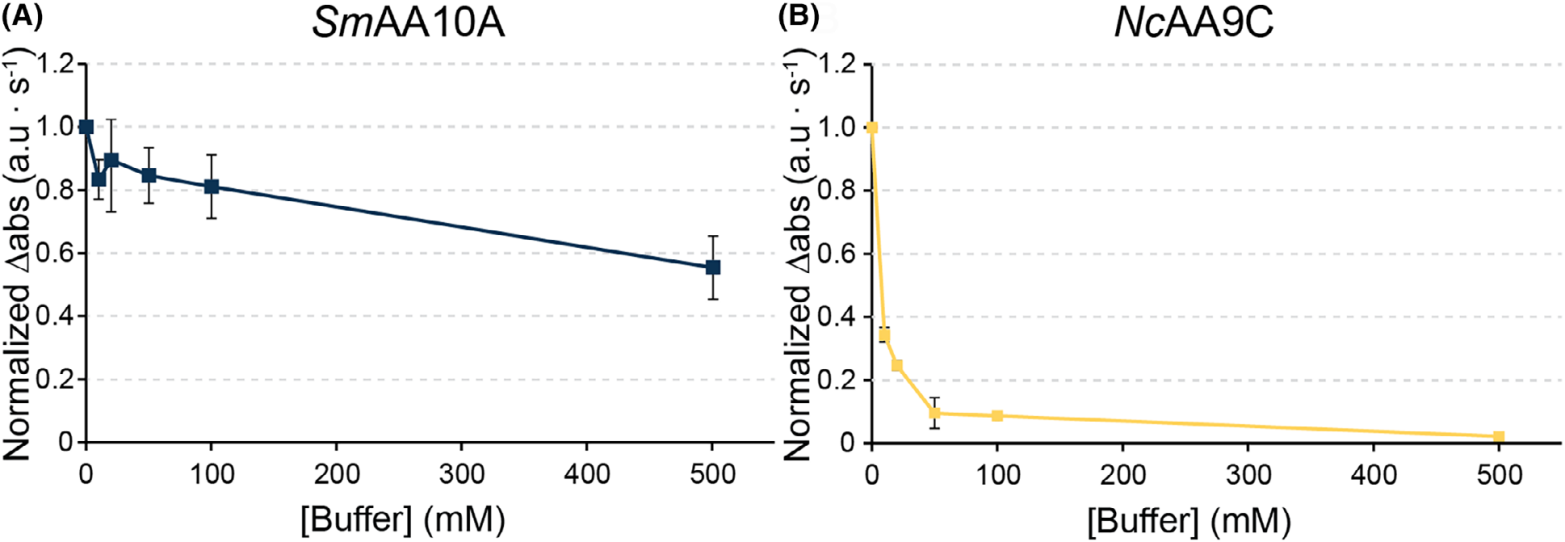
Initial rates in the 2,6‐DMP peroxidase reaction. Coerulignone formation was followed at 473 nm over time, and the linear portions of the reaction progress curves were used to determine the initial reaction rates, which were normalized. Reactions with *Sm*AA10A (A) and *Nc*AA9C (B) were prepared in 50 mm MOPS, pH 7.0, and the concentration of sodium phosphate was varied from 0 to 500 mm, and the pH was always 7.0. All reactions were performed in triplicates and standard deviations are presented as error bars for *n* = 3.

Considering the huge difference between the two LPMOs in terms of the inhibition of reduction by cyanide (Fig. 2F), a negatively charged inhibitor, we then turned to the most prevalent charged molecule during LPMO reactions, namely the buffer molecule. Thus, we assessed the effect of sodium phosphate and different positively charged buffer molecules, using stopped‐flow to monitor the effects on reduction (Fig. 7A,B), an electrochemical sensor to measure the effects on H_2_O_2_ consumption in a reaction with the substrate (Fig. 7C,D) and the 2,6‐DMP assay (Fig. 8).

The reduction rates for *Sm*AA10A show modest buffer‐dependent differences, with MOPS and Bis‐Tris giving the highest reduction rates, followed by Tris/HCl and sodium phosphate (Fig. 7A; rates given in Table S2). Although there are differences in the reduction rate, these differences are limited and all rates far exceed the *k*_cat_ for the *Sm*AA10A peroxygenase reaction with chitin (6.7 s^−1^; [68]). It would thus seem that the variation in reduction rates will not significantly affect reactions with the substrate, as was indeed observed when monitoring the consumption of H_2_O_2_ in a reaction with β‐chitin (Fig. 7C). All reactions showed similar initial rates (Fig. S8) and minimal differences in the progress curves (Fig. 7C).

While buffer effects for positively charged buffers were of similar magnitude for *Nc*AA9C, strikingly, and in stark contrast to the above, reactions in sodium phosphate showed a 10‐fold decrease in the reduction rate compared to MOPS (Fig. 7B). This, again, shows a big difference between the two LPMOs, in terms of the interaction of the copper site with negatively charged compounds. While such a buffer‐dependent difference could become visible at low reductant or substrate concentrations (see above), the difference was not visible in the initial rates obtained from the H_2_O_2_ consumption assay (Fig. 7D), which were similar for all four buffers (Fig. S9). This assay was done with xyloglucan, which is an excellent soluble substrate for *Nc*AA9C [27], with a more polymeric nature than cellopentaose. Xyloglucan could be used because the reaction is monitored by measuring H_2_O_2_ consumption, rather than by quantification of oxidized products, which is not possible for xyloglucan due to lack of standards.

Importantly, the H_2_O_2_ consumption assays with *Nc*AA9C revealed another, unexpected difference between the buffers. The reactions in sodium phosphate suffered from fast enzyme inactivation, as suggested by the early flattening of the progress curve and the failure to consume all the H_2_O_2_ (Fig. 7D). Control reactions in which fresh enzyme was added after the termination of H_2_O_2_ consumption confirmed that enzyme inactivation indeed did occur, while the reaction mixture still contained both H_2_O_2_ and reductant (Fig. S10). It is conceivable that this early inactivation relates to phosphate being a poor OH^•^ radical quencher compared to the organic buffers. Indeed, a Fenton‐like reaction in each of the tested buffers using the OH^•^ specific radical trap terephthalic acid to detect OH^•^ formation, only showed such formation for the reaction in sodium phosphate (Fig. S11).

To assess whether the observed differential impact of phosphate is specific for reactions with ascorbic acid, we also performed the 2,6‐DMP peroxidase assay, which does not employ ascorbate. Figure 8 shows a trend similar to that observed during LPMO reduction (Fig. 7A,B): increasing the phosphate concentration had only a modest effect on the activity of *Sm*AA10A whilst *Nc*AA9C was severely affected.

## Discussion

**Fig. 9:**
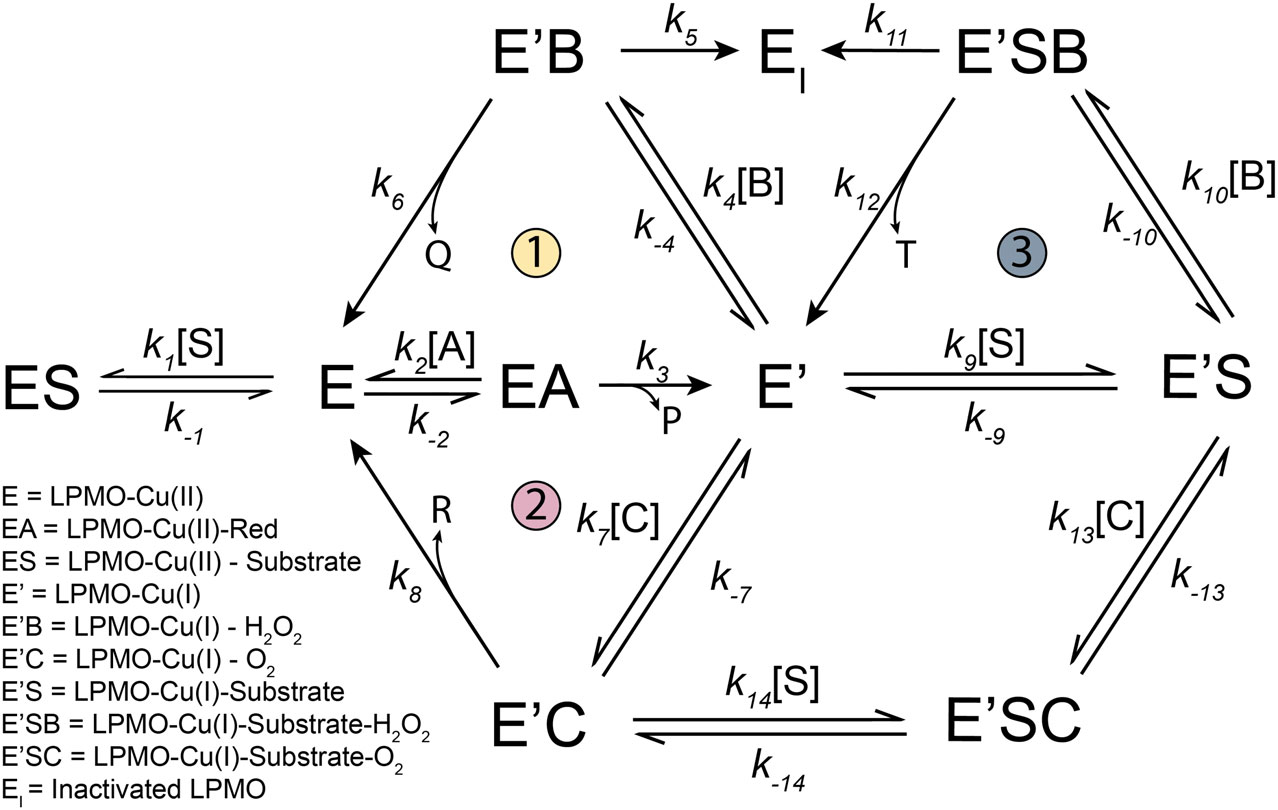
King‐Altman representation of LPMO catalysis. Double‐sided arrows indicate reversible reactions, and single‐sided arrows indicate non‐reversible reactions. All second‐order rates are converted to pseudo‐first‐order rates by adding the substrate concentrations (i.e., *k*_2_[A]). Both substrate and product inhibition are disregarded, and enzyme‐product complexes are omitted. During the LPMO reaction cycle, the copper active site is reduced from the resting Cu(II) state (E) to the active Cu(I) state (E'), which in turn can react with either O_2_ (E'C, Path 1) or H_2_O_2_ (E'B, Path 2) in a substituted enzyme reaction [74, 82]. In addition, LPMOs perform oxidative catalysis by binding both the polysaccharide substrate and H_2_O_2_ in a ternary complex reaction (E'SB, Path 3). The relative levels of different enzyme species will be affected by the presence of inhibitors such as cyanide. The reactants include A, B, C, and S, which are listed in the legend, and the various reactions lead to products P, Q, R, and T, which are oxidized reductant, water, H_2_O_2_ and oxidized substrate respectively.

The results presented above show that cyanide inhibits LPMO catalysis. While this as such is not a surprising result, our exploration of cyanide inhibition revealed several important features of LPMO catalysis as well as potential pitfalls in the functional characterization of LPMOs. We show that cyanide inhibition may be easily overlooked, depending on the reaction conditions used, and we find a remarkable difference between an AA9 and an AA10 LPMO in terms of how well the enzyme interacts with the inhibitor. In addition, more or less serendipitously, we discovered that buffer ions may have huge effects on LPMO catalysis that, again, differ between LPMO types. The present results highlight the importance of understanding the multiple factors that govern LPMO catalysis. LPMOs require three reactants to perform a catalytic turnover, the reductant, the co‐substrate, and the polysaccharide substrate, but can also engage in multiple off‐pathway reactions and may suffer from oxidative damage. The concentrations of the various substrates dictate the prevalence of reaction pathways and the balance of simultaneously occurring reactions, as illustrated by a King‐Altman plot, adapted from [32], in Fig. 9.

The basic characterization of *Sm*AA10A and *Nc*AA9C shown in Fig. 2A–E is not novel [27, 32, 61, 74], but comparing literature values can prove challenging due to varying laboratory conditions. Here the two enzymes were compared using the same methods and experimental conditions. Generally, the data (oxidase activity, rates of reduction by ascorbate and oxidation by H_2_O_2_, and activity in the 2.6‐DMP assay) and the observed differences between the two LPMOs correspond well to literature data. *Sm*AA10A has a higher reduction rate and a lower oxidation rate with both O_2_ and H_2_O_2_ compared to *Nc*AA9C in line with the higher reduction potential of the former [19, 65, 75]. The differences are likely not solely driven by the reduction potential as interactions with ascorbate, O_2_, and H_2_O_2_ will also play a role. For example, analyzing the ascorbate peroxidase reaction for both *Nc*AA9C and *Sm*AA10A. Kuusk et al. showed that the enzymes have different *K*_m (AscA)_ and $K_{m\left(H_{2}O_{2}\right)}$ values [74]. As for H_2_O_2_ in the peroxidase reaction, *Sm*AA10A has a $K_{m\left(H_{2}O_{2}\right)}$ of 60 ± 34 μm, whereas *Nc*AA9C has a $K_{m\left(H_{2}O_{2}\right)}$ of 139 ± 7 μm [74], while we show that the second‐order rate constant for oxidation of reduced *Nc*AA9C by H_2_O_2_ is some 10 times higher for the latter enzyme.

Our work with cyanide provides further insight into the differences between the two enzymes. The stopped‐flow analyses of reduction clearly showed that cyanide inhibits reduction by ascorbate, suggesting binding of cyanide to the Cu(II). The EPR spectra of both *Sm*AA10A and *Nc*AA9C in the presence of cyanide confirmed Cu(II) – CN coordination. Similar changes in EPR spectra upon addition of cyanide, i.e., an increase in *A*_||_ and resolved nitrogen superhyperfines, have been observed for other copper enzymes such as Cu‐Zn superoxide dismutase [76], dopamine – β‐monooxygenase [49, 77] and galactose oxidase [78, 79]. In addition, EPR spectra collected for an AA11 LPMO in the presence of azide also show a direct coordination with similar effects to *A*_||_ [80]. Importantly, the EPR data show that cyanide binds to the Cu(II) state of the enzyme, in line with the notions that cyanide is a mimic of superoxide [76, 81] and that LPMOs form Cu(II)‐superoxide like intermediates [50, 52]. The effects on reduction rates show that cyanide binds much stronger to *Nc*AA9C‐Cu(II) compared to *Sm*AA10A‐Cu(II).

To understand the observed impacts of cyanide one needs to consider several of the concentrations and rates shown in the King‐Altman plot of Fig. 9, as alluded to above. For example, the lack of cyanide inhibition in *in situ* H_2_O_2_‐limiting reactions for both enzymes is due to LPMO reduction not being rate‐limiting. The theoretical reduction rates in the presence of the added surplus of ascorbate (1 mm) are in the order of 520 and 170 s^−1^ (calculated from the second‐order rates provided in Tables S2 and S3) for *Sm*AA10A and *Nc*AA9C, respectively. The rate of *in situ* H_2_O_2_‐limiting LPMO reactions is on the min^−1^ scale [40, 69] and will, therefore, not be limited by inhibition of reduction by cyanide. On the other hand, in reactions with exogenously added H_2_O_2_, the lower ascorbate concentration (100 μm) leads to expected reduction rates of 52 and 17 s^−1^ for *Sm*AA10A and *Nc*AA9C respectively. These reduction rates are close to reported enzymatic turnover numbers for H_2_O_2_‐driven reactions [27, 68, 69], and, thus, cyanide inhibition becomes detectable. The type and concentration of the substrate also play major roles. If there is plenty, easily accessible substrate, the LPMO remains in the active Cu(I) state. The reaction will predominately follow path 3 (Fig. 9), whereas reoxidation of the enzyme to the Cu(II) state, the re‐reduction of which would be inhibited by cyanide, is avoided. At lower effective substrate concentrations, paths 1 and 2 (oxidase and peroxidase side reactions, Fig. 9), each including a reduction reaction, will become more prominent, and the LPMO becomes susceptible to cyanide inhibition. The studies of the dependency of cyanide inhibition on the reductant concentration (Fig. 6) and the type (Fig. 5) and concentration (Fig. 6) of substrate support these considerations.

In some of the experiments described above, the presence of cyanide had unexpected minor positive effects on product formation. Further work is needed to rigorously explain these results, however two interesting and interconnected possible underlying causes stand out. Firstly, many of these observations can be explained by assuming that cyanide binding promotes the oxidase activity of LPMOs. Increased H_2_O_2_ production due to increased oxidase activity could explain the increased product yield. So far, the rate‐limiting step in LPMO‐catalyzed production of H_2_O_2_ has not been established and, besides, two reaction scenarios are being considered. In one of these, the superoxide resulting from reduction of O_2_ by the LPMO‐Cu(I) is released from the enzyme, followed by spontaneous disproportionation or a reductant‐driven reaction to yield H_2_O_2_. Alternatively, H_2_O_2_ can be generated directly at the copper site, which would require the supply of a second electron and two protons to the LPMO‐Cu(II)‐superoxide complex [83]. It is tempting to speculate that cyanide, being a superoxide mimic [76, 81], could displace the Cu(II) bound superoxide and thus promote formation of H_2_O_2_ through the first scenario. This would imply that superoxide release is a rate‐limiting step in the oxidase reaction.

The experimental data support this scenario, i.e., cyanide promoting release of superoxide from the LPMO, quite well. In *in situ* H_2_O_2_‐limiting reactions, i.e. reactions that to a considerable extent depend on oxidase activity, cyanide leads to slightly increased product formation for both LPMOs (Figs 4A and 5A). This effect is stronger for *Nc*AA9C, which is in perfect accordance with the stronger binding of cyanide for this enzyme. Figure 5A shows that increased initial rates in the presence of cyanide are accompanied by early onset of enzyme inactivation, which is typical for *in situ* H_2_O_2_‐limiting reactions in which too much H_2_O_2_ is generated. Other evidence for the positive impact of cyanide on the oxidase activity comes from Fig. 5B showing degradation of cellopentaose in the presence of exogenously added H_2_O_2_. At 200 and 400 μm cyanide, the product levels of this reaction exceed the amount of added H_2_O_2_, which can only be explained by the *in situ* formation of additional H_2_O_2_. Of note, in this case product formation increases gradually over time, which is nicely compatible with the time scale of the *in situ* H_2_O_2_ limited reaction depicted in Fig. 5A.

Secondly, other effects may be at play, and promotion of the oxidase activity by cyanide cannot explain all the unexpected minor effects of cyanide on product formation. In several of the reactions, enzyme inactivation, which is accompanied by copper release [42], will take place. The release of free copper into a reductant‐containing reaction mixture may lead to all sorts of reactions, including additional LPMO inactivation [43]. We suspect that some of the observed and not yet discussed unexpected cyanide effects relate to the proven (Fig. S5) impact of cyanide on the reactivity of free copper.

The higher affinity of *Nc*AA9C for cyanide could potentially relate to an important difference in the second spheres of the two LPMOs studied here. A key second sphere residue in *Sm*AA10A is a negatively charged glutamate known to coordinate H_2_O_2_ [18], whilst *Nc*AA9C contains a neutral glutamine in an equivalent position [19]. It is conceivable that a negatively charged glutamate close to the active site repulses negatively charged cyanide, which could help explain why cyanide binds better to *Nc*AA9C.

Interestingly, the higher affinity *Nc*AA9C for cyanide was transferable to the negatively charged phosphate ion. When present at standard buffer concentrations, phosphate gave a 10‐fold decrease in the reduction rate and led to inhibition in the ascorbate‐independent 2,6‐DMP peroxidase reaction. The lack of inhibition observed for *Sm*AA10A by sodium phosphate again points towards different electronic structures in the active site. It is remarkable and important that commonly used buffers have such a strong impact on the functionality of *Nc*AA9C.

Most remarkably, the experiments with various buffers revealed a huge effect of the buffer ion on the stability of *Nc*AA9C in turnover conditions. Apparently, in phosphate buffer, *Nc*AA9C is much more vulnerable to oxidative damage than in organic buffers. Organic buffers can react with radicals such as OH^•^, thus removing damaging oxidants from the solution [84, 85]. On the other hand, sodium phosphate is commonly used in radical spin trapping experiments due to a lack of reactivity with radicals [86]. Control reactions with an OH^•^ quencher indeed showed that OH^•^ only accumulated in reactions with sodium phosphate. It may thus seem that the buffer affects radical formation and/or how well LPMOs can deal with such radicals. Perhaps, the buffer affects the efficiency of protective hole hopping pathways, that, notably, seem to differ between *Sm*AA10A [72] and AA9 type LPMOs [87]. These intriguing observations, including the remarkable difference between *Sm*AA10A and *Nc*AA9C, warrant further studies and should serve as a cautionary tale for future LPMO research.

In conclusion, to the best of our knowledge, this is the first LPMO inhibition study presenting a quantitative investigation of both off‐pathway and on‐pathway LPMO reactions for two different LPMOs. The results and considerations presented above showcase the complexity of LPMO catalysis and the importance of understanding limiting factors during LPMO reactions. We show that cyanide binds the resting Cu(II) state LPMO, which results in inhibition during reaction conditions that involve formation of the Cu(II) state and that are limited by reduction of the enzyme. The complexity of LPMO reactions is due to the occurrence of several interconnected reactions at the same time, which are governed by the availability of the reductant, O_2_, H_2_O_2_ and the polysaccharide substrate. Our results reveal remarkable differences between the two studied LPMOs and it will be interesting to see if these differences are generally applicable to families of LPMOs with similar active site architectures. Finally, the here discovered LPMO‐dependent large effects of the buffer ion on LPMO functionality warrant deeper and wider follow‐up studies that will be of major importance to the field.

## Author contributions

OG, LS, ZF, AAS and VGHE conceptualized and planned the experiments. OG, LS, TZE‐M performed the experiments. OG, LS, ZF, AAS, TZE‐M, KRH, IA‐F, VGHE and ÅKR interpreted and discussed the data. OG and VGHE prepared the manuscript draft. MS and RL interpreted data. All authors discussed, edited and proof‐read the manuscript.

### Peer review

The peer review history for this article is available at https://www.webofscience.com/api/gateway/wos/peer‐review/10.1002/1873‐3468.15105.

## Supporting information


**Fig. S1.** Workflow and data treatment when measuring H_**2**_O_**2**_ consumption with the electrochemical sensor.
**Fig. S2.** Stopped‐flow control reactions comparing LPMO reduction with ascorbate or cyanide.
**Fig. S3.** Cyanide as a possible reductant in *Sm*AA10A reactions.
**Fig. S4.** H_2_O_2_ stability in the presence of KCN.
**Fig. S5.** Effect of cyanide on the activity of *Sm*AA10A in *in situ* H_2_O_2_ ‐limiting reactions that contain free copper.
**Fig. S6.** Product formation by *Sm*AA10A.
**Fig. S7.** Inhibition of 2,6‐DMP oxidation by cyanide.
**Fig. S8.** Turnover numbers for *Sm*AA10A acting on β‐chitin in various buffers.
**Fig. S9.** Turnover numbers for *Nc*AA9C acting on xyloglucan in various buffers.
**Fig. S10.** H_2_O_2_ consumption by *Nc*AA9C acting on xyloglucan in a phosphate buffer monitored with an electrochemical sensor.
**Fig. S11.** Formation of OH^•^ radicals in various buffers.
**Table S1.** Spin Hamiltonian parameters of LPMOs in the absence and presence of cyanide.
**Table S2.** Second‐order reduction rates for *Sm*AA10A in varying buffers (50 mM, pH 7.0) with 100 μM ascorbate.
**Table S3.** Second‐order reduction rates for *Nc*AA9C in varying buffers (50 mM, pH 7.0) with 100 μM ascorbate.

## Data Availability

All underlying raw data is available in a public repository: https://doi.org/10.18710/EBZKZQ.
