# Supplementary material for: Functional variation among LPMOs revealed by the inhibitory effects of cyanide and buffer ions
Source: FEBS Lett. 2025 Feb 6;599(9):1317–36. doi: 10.1002/1873-3468.15105 (PMC12067858; doi:10.1002/1873-3468.15105)
Supplement: Supplementary file 1 — Fig. S1. Workflow and data treatment when measuring H 2 O 2 consumption with the electrochemical sensor. Fig. S2. Stopped‐flow control reactions comparing LPMO reduction with ascorbate or cyanide. Fig. S3. Cyanide as a possible reductant in SmAA10A reactions. Fig. S4. H2O2 stability in the presence of KCN. Fig. S5. Effect of cyanide on the activity of SmAA10A in in situ H2O2 ‐limiting reactions that contain free copper. Fig. S6. Product formation by SmAA10A. Fig. S7. Inhibition of 2,6‐DMP oxidation by cyanide. Fig. S8. Turnover numbers for SmAA10A acting on β‐chitin in various buffers. Fig. S9. Turnover numbers for NcAA9C acting on xyloglucan in various buffers. Fig. S10. H2O2 consumption by NcAA9C acting on xyloglucan in a phosphate buffer monitored with an electrochemical sensor. Fig. S11. Formation of OH• radicals in various buffers. Table S1. Spin Hamiltonian parameters of LPMOs in the absence and presence of cyanide. Table S2. Second‐order reduction rates for SmAA10A in varying buffers (50 mM, pH 7.0) with 100 μM ascorbate. Table S3. Second‐order reduction rates for NcAA9C in varying buffers (50 mM, pH 7.0) with 100 μM ascorbate. [file FEB2-599-1317-s001.docx]

**Supplementary information**

**Functional variation among LPMOs revealed by the inhibitory effects of cyanide and buffer ions**

Ole Golten^a^, Lorenz Schwaiger^b^, Zarah Forsberg^a^, Kelsi R. Hall^a,c^, Anton A. Stepnov^a^, Tom Z. Emrich-Mills^a^, Iván Ayuso Fernández^a,d^, Morten Sørlie^a^, Roland Ludwig^b^, Åsmund Kjendseth Røhr^a^, Vincent G.H. Eijsink^a^*

^a^: Faculty of Chemistry, Biotechnology and Food Science, Norwegian University of Life Sciences (NMBU), P.O. box 5003, 1430 Ås, Norway

^b^: Department of Food Science and Technology, Institute of Food Technology, University of Natural Resources and Life Sciences, Vienna (BOKU), Muthgasse 18, 1190 Vienna, Austria

^c^: School of Biological Sciences, University of Canterbury, Christchurch 8140, New Zealand

^d^: Biotechnology Department, Margarita Salas Center for Biological Research (CIB-CSIC), Madrid, 28040, Spain

*Corresponding author: Vincent G. H. Eijsink, vincent.eijsink@nmbu.no


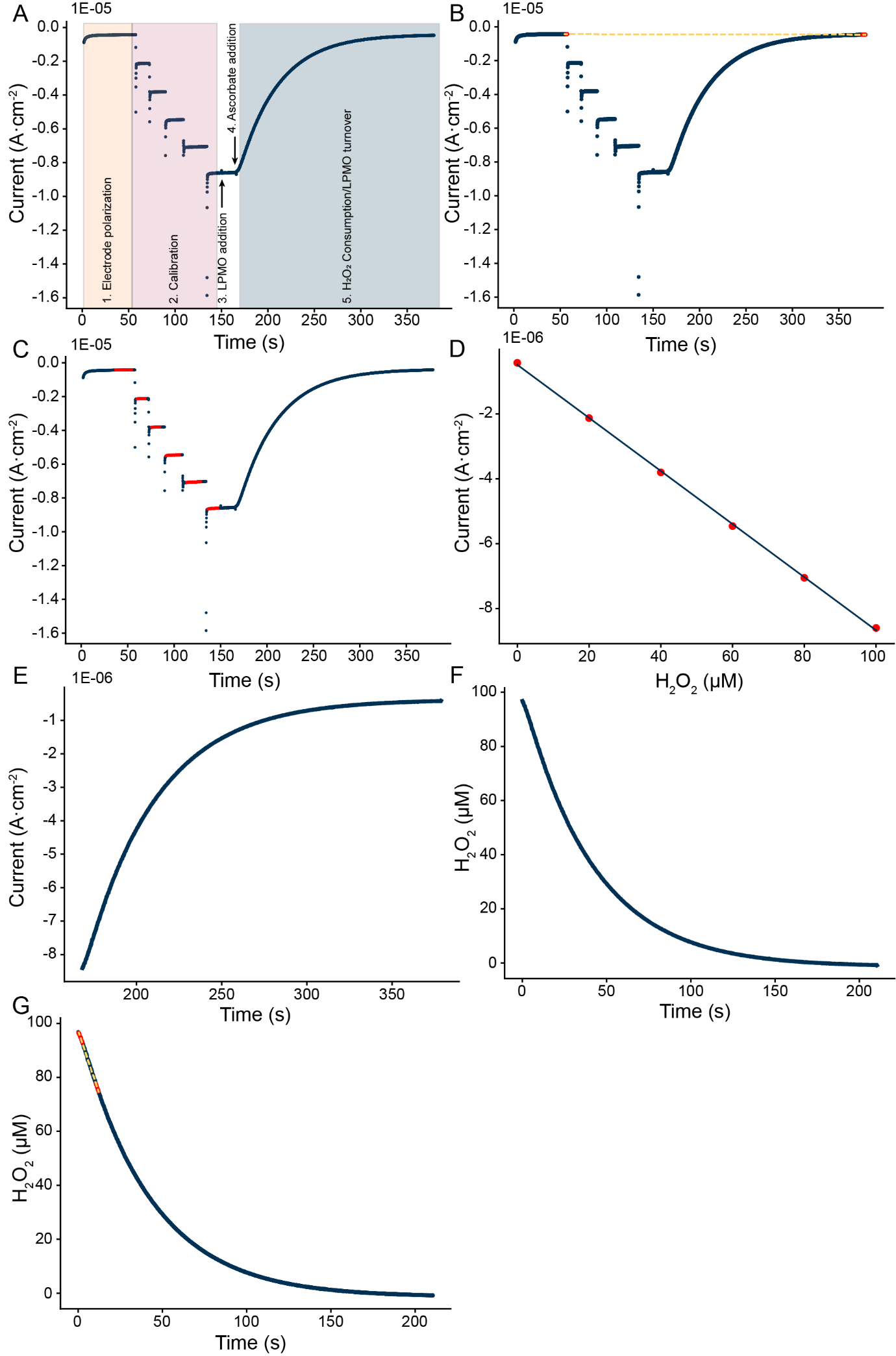


**Figure S1: Workflow and data treatment when measuring H_2_O_2_ consumption with the electrochemical sensor.** For a more extensive explanation and discussion, please refer to the original publication [1]. (A) Raw data trace collected using the NOVA 2 software (Metrohm, Herisau, Switzerland) with the electrochemical setup described in the Materials and Methods section. The reaction consists of 1) electrode polarization in the substrate and buffer solution allowing for a stable baseline, 2) Five stepwise additions of 20 μM H_2_O_2_ resulting in a decrease in current, 3) addition of LPMO, 4) addition of ascorbate initiating the LPMO reaction and 5) the decrease of H_2_O_2_ due to LPMO turnover. (B) Baseline correction of instrument drift was performed by performing a linear regression (yellow line) between two points, one being the last part of the electrode polarization step (red points, 60 seconds) and the other at the end of the data collection (red points, 360 seconds). The slope of the linear regression was used to correct the current values. (C) Internal calibration of the Prussian blue modified gold working electrode was performed by adding H_2_O_2_ in five steps of either 10 or 20 µM to a final concentration of either 50 or 100 µM. An average (red points) of the values in each data plateau was used to determine the current (nA) for each concentration of H_2_O_2_. (D) A standard curve was created by plotting current (nA) vs H_2_O_2_ (µM). (E) Selection of the start of the reaction was performed by manually selecting the point where ascorbate was added to start the reaction and the data trace was truncated to only contain values for the LPMO reaction. (F) The data was then converted from current (nA) to H_2_O_2_ (µM), using the calibration curve shown in panel D, and the time was corrected to show the LPMO reaction start at t = 0. (G) The initial rate of the reaction was calculated from the initial linear portion of the H_2_O_2_ consumption curve. Each data treatment step explained above was manually inspected using an in-house generated command line interface available for all on Github ([GitHub - ogo001/H2O2_RDE](https://github.com/ogo001/H2O2_RDE)).


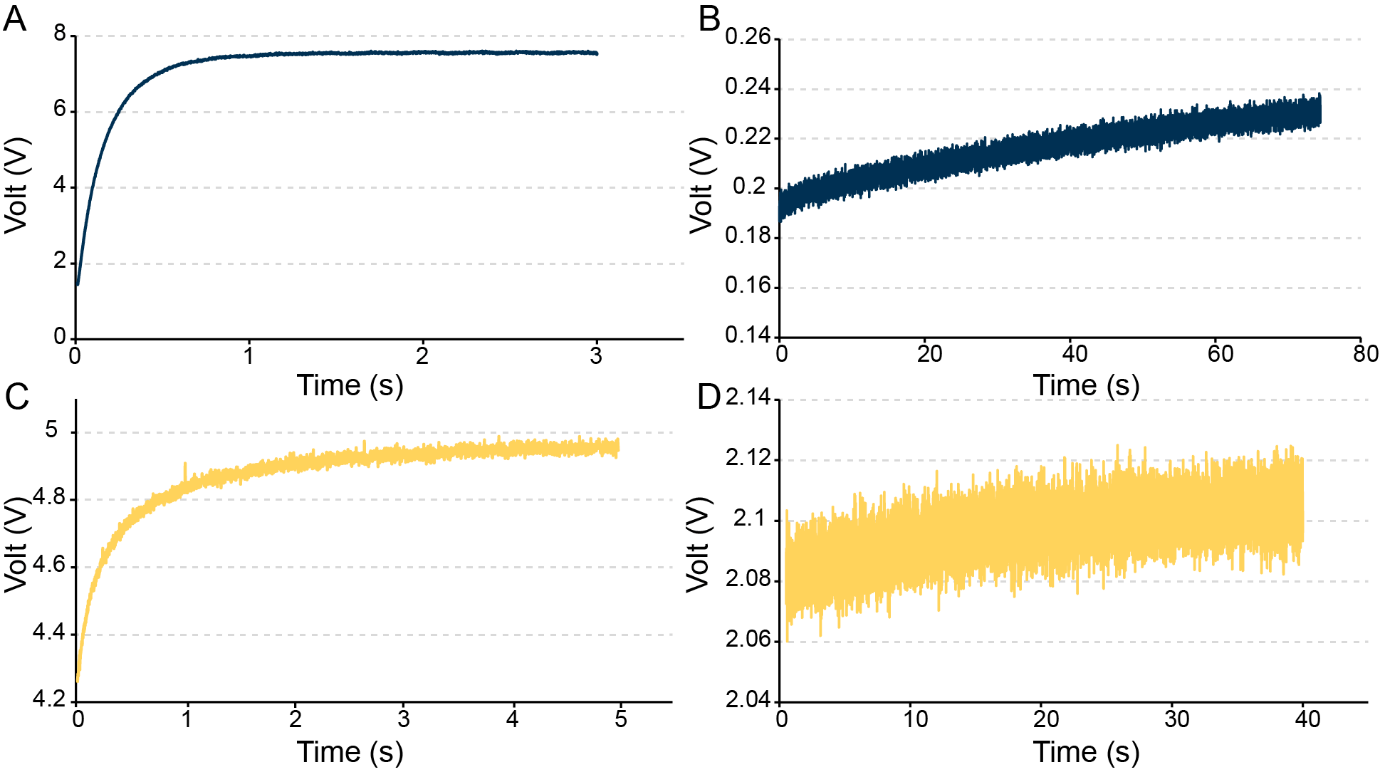


**Figure S2: Stopped flow control reactions comparing LPMO reduction with ascorbate or cyanide.** Reduction, reflected in a change in fluorescence (here expressed as photomultiplier voltage), was measured by mixing 10 µM SmAA10A (A,B) or NcAA9C (C,D) with either 10 µM ascorbate using single mixing (A,C) or 1 mM cyanide using double mixing (B, D). All reactions were performed in 50 mM MOPS, pH 7.0, in triplicates (not shown). The graphs clearly show that cyanide does not reduce the LPMOs.


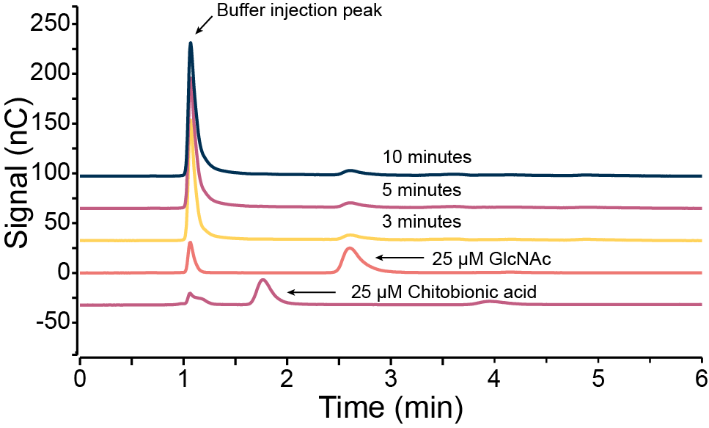


**Figure S3: Cyanide as a possible reductant in SmAA10A reactions.** Investigation of the reducing power of cyanide was performed by adding 200 µM cyanide instead of ascorbate to reactions containing 1 μM SmAA10A, 10 g L^-1^ β-chitin, 200 μM H_2_O_2_. Reactions were terminated at different time points (indicated in the figure) by filtering through a 0.45 um filter, before treating the filtrate with chitobiase (SmCHB) to convert the different oligosaccharides to the oxidized dimer (chitobionic acid) and the native monomer (NAcGlc), which simplifies analysis and quantification of the oxidized dimer. The graphs show that no oxidized products were formed, which means that cyanide cannot reduce the LPMO. The peak at the 1-minute mark is the buffer injection peak which contains all components which do not interact with the column material and is therefore higher for the enzymatic reactions than the standards.


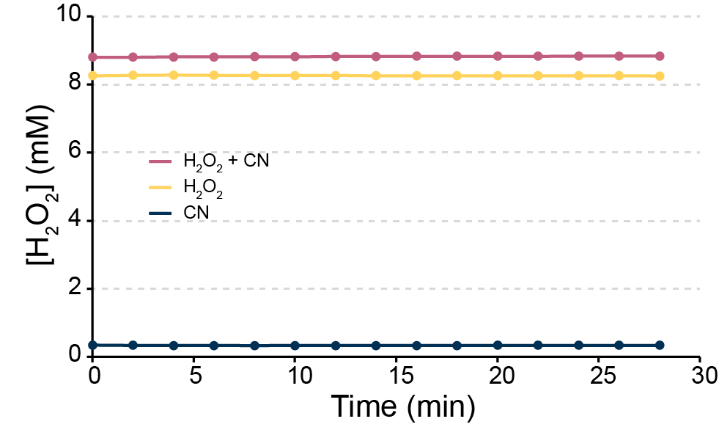


**Figure S4: H­_2_O_2_ stability in the presence of KCN.** The concentration of H_2_O_2_ was determined by monitoring the absorbance at 240 nm using the molar extinction coefficient of 43.6 cm^-1^·M^-1^. The reactions were carried out by adding 900 µL of a 10 mM H_2_O_2_ stock solution into a quartz cuvette followed by adding either 100 µL ddH_2_O_2_ (yellow) or 100 µL 100 mM KCN (pink) and subsequent monitoring of the absorbance over time. The increased absorbance values observed for the samples with both H_2_O_2_ and KCN are due to the contribution of KCN alone at 240 nm (blue).


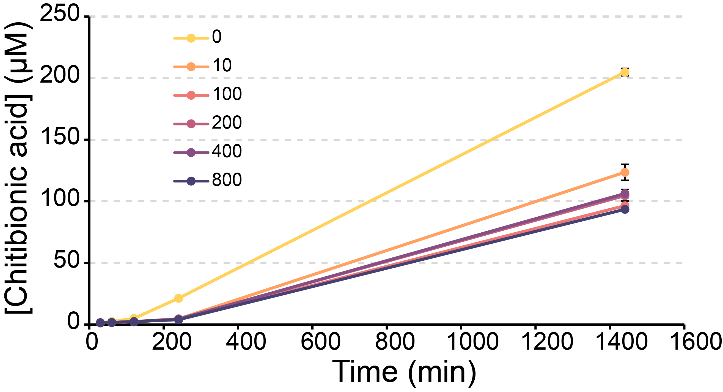


**Figure S5: Effect of cyanide on the activity of SmAA10A in in situ H_2_O_2 ­­_-limiting reactions that contain free copper.** Chitin degradation by SmAA10A was performed in 50 mM MOPS, pH 7.0, with 10 g·L^-1^ β-chitin, 1 mM ascorbate, 1 µM SmAA10A, 1 µM CuSO­_4_ and different concentrations of cyanide (0-800 µM). Reactions were terminated by filtering through a 0.45 um filter, before treating the filtrate with chitobiase (SmCHB) to degrade the different oligosaccharides to the oxidized dimer (chitobionic acid) and the native monomer (GlcNAc)) to simplify analysis and quantify the oxidized dimer. All reactions were performed in triplicates and the standard deviations are reported as error bars (n=3). Under these conditions, free copper promotes in situ generation of H_2_O_2_ through oxidation of ascorbate.


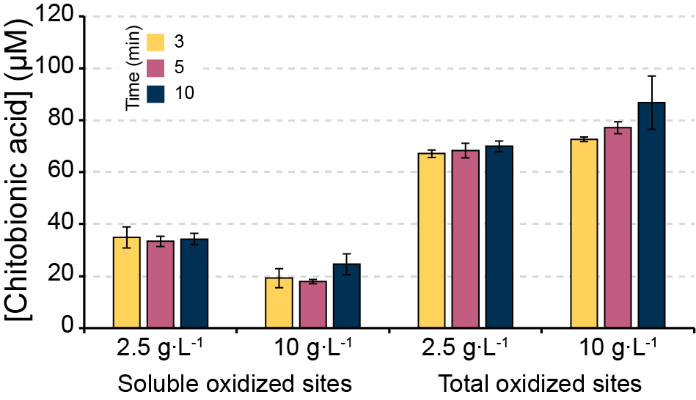


**Figure S6: Product formation by SmAA10A.** Reaction mixtures consisted of 1 µM SmAA10A, 50 mM MOPS, pH 7.0, 2.5 or 10 g·L^-1^ β-chitin, 100 µM H_2_O_2,_ and the reactions were initiated by adding 100 µM ascorbate. Soluble oxidized products were quantified by filtering 75 µL sample through a 0.45 µm filter at 3, 5 and 10 minutes followed by addition of 1 µM SmCHB to degrade the soluble chitooligomers to the oxidized dimer and native monomer. For the determination of total oxidized sites, 75 µL of sample was boiled for 10 minutes to deactivate the LPMO followed by a 2-fold dilution and addition of SmChiA and SmCHB (1 µM of each; final concentration). The subsequent degradation reaction was performed at 40 °C for 24 hours with 850 rpm agitation, followed by filtering the mixture through a 0.45 µm filter. For both reaction setups, the oxidized dimer was quantified using an Ultimate 3000 Dionex UHPLC equipped with a 100 x 7.8 Rezex RFQ-Fast acid H+ (8%) column. All reactions were performed in triplicates and the standard deviations are reported as error bars (n=3)


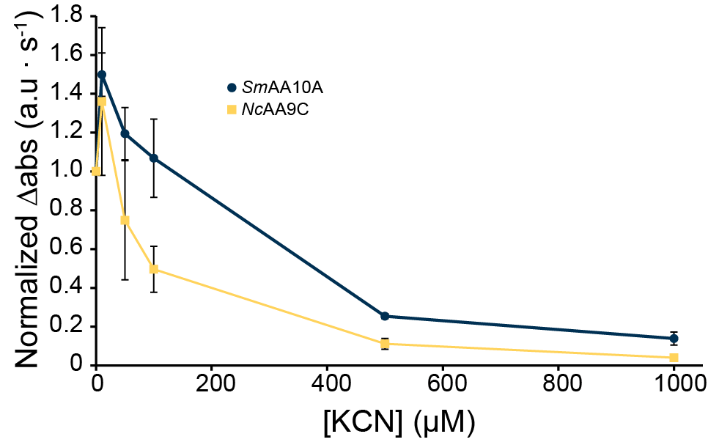


**Figure S7: Inhibition of 2,6-DMP oxidation by cyanide.** The initial rate of the 2,6-DMP oxidation reaction was monitored by measuring the absorbance at 473 nm, which reflects the formation of coerulignone, for 2 µM SmAA10A (blue) and 2 µM NcAA9C (yellow) in 50 mM MOPS, pH 7.0 containing varying amounts of KCN. The insert shows a zoomed enlargement of the 0 - 100 µM KCN region, to accentuate the boost in activity at low cyanide concentration. All reactions were performed in triplicates and the standard deviations are reported as error bars (n=3)


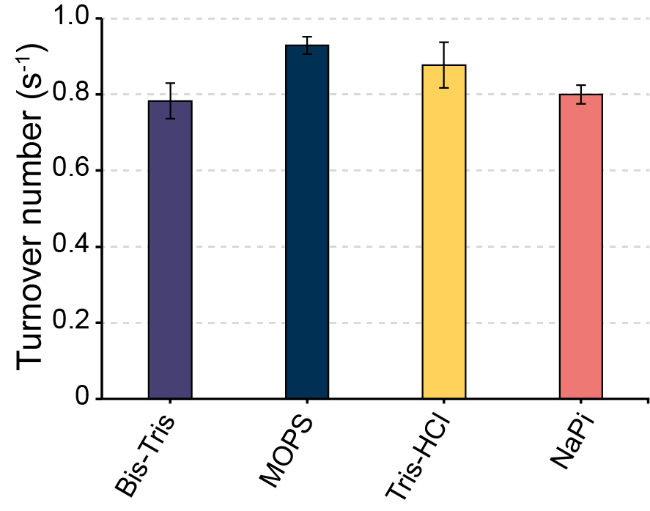


**Figure S8: Turnover numbers for SmAA10A acting on β-chitin in various buffers.** Turnover numbers for SmAA10A were determined by monitoring H­_2_O_2_ consumption in a reaction with 1 µM enzyme acting on 10 g L^-1^ β-chitin suspended in 50 mM buffer, pH 7.0, containing 50 μM H_2_O_2_ and 100 mM KCl as a supporting electrolyte. The turnover numbers were calculated from the linear parts of the H_2_O_2_ consumption curves shown in Figure 7C using the following equation $\text{TN} (s^{-1})= \frac{v_{0} (\mu M s^{-1})}{\left[ E \right](\mu M)}$. Standard deviations are shown as error bars for n =3.


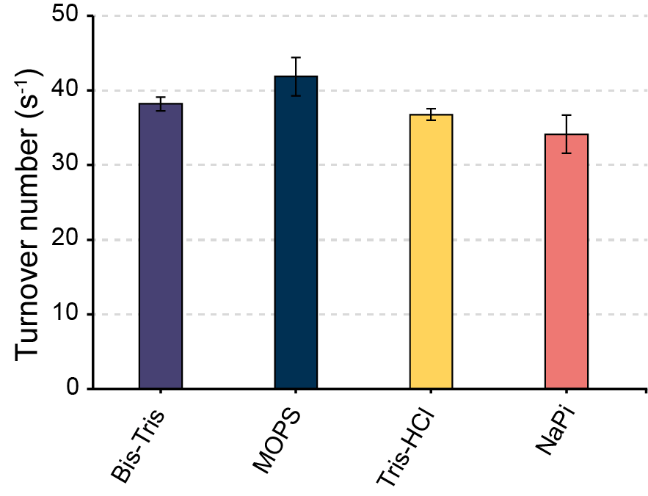


**Figure S9: Turnover numbers for NcAA9C acting on xyloglucan in various buffers.** Turnover numbers for NcAA9C were determined by monitoring the consumption of H­_2_O_2_ in a reaction with 50 nM enzyme, 4 g L^-1^ xyloglucan and 100 μM H_2_O_2_ in 50 mM buffer, pH 7.0, and 100 mM KCl as a supporting electrolyte. The turnover numbers were calculated from the linear parts of the H_2_O_2_ depletion curves shown in Figure 7D using the following equation $\text{TN} (s^{-1})= \frac{v_{0} (\mu M s^{-1})}{\left[ E \right](\mu M)}$. Standard deviations are shown as error bars for n =3, except reactions in MOPS, where n = 2.


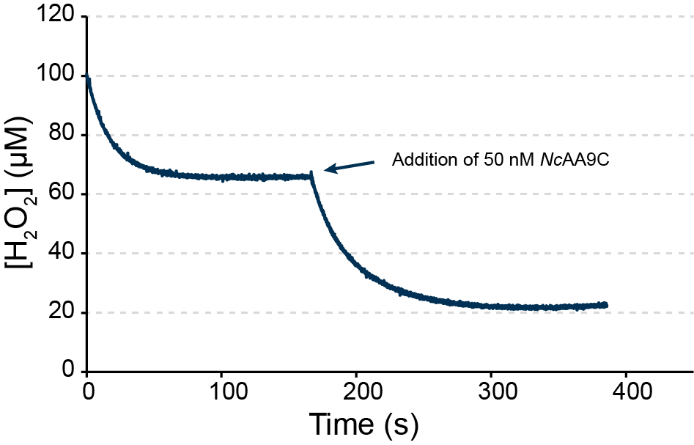


**Figure S10: H_2_O_2_ consumption by NcAA9C acting on xyloglucan in a phosphate buffer monitored with an electrochemical sensor.** The reaction mixture contained 50 nM NcAA9C, 4 g∙L^-1^ xyloglucan, 100 µM ascorbate, 100 mM KCl and 100 µM H_2_O_2_ in 50 mM sodium phosphate, pH 7.0. Under these conditions, the baseline was not reached, which is due to enzyme inactivation, as demonstrated by the impact of adding fresh NcAA9C at approximately 180 seconds.


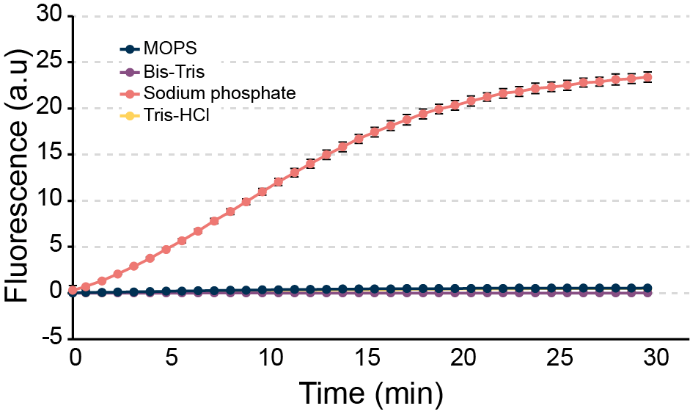


**Figure S11: Formation of OH^●^ radicals in various buffers.** Hydroxy radical formation in a Fenton-like reaction with 5 µM CuSO_4_, 1 mM ascorbate, 100 µM H_2_O_2_, 100 µM terephthalic acid and 50 mM buffer (MOPS, Bis-Tris, Tris-HCl and sodium phosphate), pH 7.0, was monitored using a 312 nm excitation wavelength and a 328 nm emission wavelength. All reactions were performed in triplicates and standard deviations are reported as error bars.

**Table S1: Spin Hamiltonian parameters of LPMOs in the absence and presence of cyanide.**

| **Parameter** | ***Sm*AA10A** | ***Sm*AA10A -CN** | ***Nc*AA9C** | ***Nc*AA9C - CN** |
| --- | --- | --- | --- | --- |
| *g_z_* | 2.255 | 2.202 | 2.272 | 2.204 |
| *g_y_* | 2.111 | 2.036 | 2.074 | 2.054 |
| *g_x_* | 2.024 | 1.999 | 2.032 | 2.035 |
| *A_z_* | 355 | 472 | 465 | 600 |
| *A_y_* | 50 | 55 | 120 | 70 |
| *A_x_* | 230 | 50 | 80 | 70 |

**Table S2. Second order reduction rates for SmAA10A in varying buffers (50 mM, pH 7.0) with 100 µM ascorbate.** These rates were determined by linear regression of the observed pseudo-first-order reaction rates versus the ascorbate concentration, as depicted in Fig. 7A of the main manuscript. Standard deviations were calculated from the regression analysis. Each reaction was performed in triplicates.

| **50 mM Buffer pH 7.0** | **Second order reduction rate (M^-1^·s^-1^)** | **Linear fit (R^2^)** |
| --- | --- | --- |
| Tris-HCl | 397 000±11 000 | 0.99 |
| Sodium phosphate | 394 000±10 000 | 0.99 |
| MOPS | 527 000±15 000 | 0.99 |
| Bis-Tris | 508 000±17 000 | 0.99 |

**Table S3. Second order reduction rates for NcAA9C in varying buffers (50 mM, pH 7.0) with 100 µM ascorbate.** These rates were determined by linear regression of the observed first-order reaction rates versus the ascorbate concentration, as depicted in Fig. 7B of the main manuscript. Standard deviations were calculated from the regression analysis. Each reaction was performed in triplicates.

| **50 mM Buffer pH 7.0** | **Second order reduction rate (M^-1^·s^-1^)** | **Linear fit (R^2^)** |
| --- | --- | --- |
| Tris-HCl | 92 000±6 000 | 0.98 |
| Sodium phosphate | 8 300±600 | 0.98 |
| MOPS | 167 000±5 700 | 0.99 |
| Bis-Tris | 129 000±7 500 | 0.99 |

**References**

1. Schwaiger, L., Csarman, F., Chang, H., Golten, O., Eijsink, V. G. H. & Ludwig, R. (2024) Electrochemical monitoring of heterogeneous peroxygenase reactions unravels LPMO kinetics, *ACS Catalysis.* **14**, 1205-1219.
